# Natural and Experimental SARS-CoV-2 Infection in Domestic and Wild Animals

**DOI:** 10.3390/v13101993

**Published:** 2021-10-04

**Authors:** David A. Meekins, Natasha N. Gaudreault, Juergen A. Richt

**Affiliations:** 1 Department of Diagnostic Medicine/Pathobiology, College of Veterinary Medicine, Kansas State University, Manhattan, KS 66506, USA; dmeekins@vet.k-state.edu (D.A.M.); nng5757@vet.k-state.edu (N.N.G.); 2 Center of Excellence for Emerging and Zoonotic Animal Diseases (CEEZAD), College of Veterinary Medicine, Kansas State University, Manhattan, KS 66502, USA

**Keywords:** SARS-CoV-2, COVID-19, zoonotic disease, coronavirus, veterinary science, virology, animal models

## Abstract

SARS-CoV-2 is the etiological agent responsible for the ongoing COVID-19 pandemic, which continues to spread with devastating effects on global health and socioeconomics. The susceptibility of domestic and wild animal species to infection is a critical facet of SARS-CoV-2 ecology, since reverse zoonotic spillover events resulting in SARS-CoV-2 outbreaks in animal populations could result in the establishment of new virus reservoirs. Adaptive mutations in the virus to new animal species could also complicate ongoing mitigation strategies to combat SARS-CoV-2. In addition, animal species susceptible to SARS-CoV-2 infection are essential as standardized preclinical models for the development and efficacy testing of vaccines and therapeutics. In this review, we summarize the current findings regarding the susceptibility of different domestic and wild animal species to experimental SARS-CoV-2 infection and provide detailed descriptions of the clinical disease and transmissibility in these animals. In addition, we outline the documented natural infections in animals that have occurred at the human–animal interface. A comprehensive understanding of animal susceptibility to SARS-CoV-2 is crucial to inform public health, veterinary, and agricultural systems, and to guide environmental policies.

## 1. Introduction

Coronavirus disease 2019 (COVID-19) is a respiratory illness caused by infection with severe acute respiratory syndrome coronavirus 2 (SARS-CoV-2), which emerged in late 2019 in Wuhan, China [1]. Over a year later, SARS-CoV-2 continues to spread worldwide and has resulted in over 200 million documented cases and 4.5 million deaths as of 1 September 2021 [2]. Mitigation strategies to control the spread of SARS-CoV-2 have mainly consisted of the implementation of social distancing policies and changes in community behavior, which have had significant socio-economic consequences [3,4]. In recent months, new therapeutic treatments have lowered the SARS-CoV-2 fatality rate and several effective vaccines have been approved and are currently being used worldwide [5,6,7,8]. However, the global scale of the pandemic and inherent complications involved with vaccination efforts to obtain herd immunity ensures that SARS-CoV-2 will be a significant feature of the global health landscape for the foreseeable future.

Coronaviruses are enveloped, single-stranded, positive-sense RNA viruses with large genomes ranging from 29 to 32 kilobases (kb) in length [9,10]. Coronaviruses belong to the order *Nidovirales* in the *Coronaviridae* family (*Orthocoronavirinae* subfamily) and are composed of four genera, based on their phylogeny and genomic structures, designated *alpha-*, *beta-*, *gamma-*, and *deltacoronavirus* [9,10,11]. So far, seven different coronaviruses have been identified that infect humans [12,13]. Infections with human coronavirus (HCoV)-OC43, HCoV-229E, HCoV-NL63, and HCoV-HKU1 generally result in mild-to-moderate respiratory disease and are responsible for up to 30% of common colds [10,12,13]. In contrast, SARS-CoV, MERS-CoV, and SARS-CoV-2, all of which emerged in the 21st century, can cause severe fatal respiratory disease [10,12,13]. SARS-CoV-2 is a *betacoronavirus* and is most closely related to SARS-CoV, with 79.6% genetic similarity [1].

SARS-CoV-2 elicits a wide spectrum of clinical disease manifestations and has achieved sustained human-to-human transmission [14]. Most individuals infected with SARS-CoV-2 remain asymptomatic or develop mild-to-moderate disease symptoms, including fever, cough, dyspnea, fatigue, and anosmia. In approximately 20% of cases, the disease can extend into the lower respiratory tract, resulting in pneumonia, among which about 5% of these cases progress into acute respiratory distress syndrome (ARDS) [15,16]. Ground-glass opacities in the lungs are detected using computed tomography (CT) scans of patients suffering from severe COVID-19, and histological examinations reveal pulmonary edema and alveolar damage. Moreover, some COVID-19 patients suffer from organ damage, including kidney, liver, and cardiac complications. Age and sex are primary predictors of mortality, along with comorbidities that include chronic pulmonary/cardiovascular disease, obesity, and diabetes [15,16]. Transmission of SARS-CoV-2 mainly occurs via respiratory droplets and aerosols, and asymptomatic individuals are capable of transmission [14,17]. The contribution of fomite transmission is still debated [18,19].

Coronaviruses infect a wide range of host species, including cats, pigs, ferrets, rabbits, rats, birds, cattle, and horses [20,21]. Different coronaviruses have been demonstrated to cross species barriers and adapt to new hosts [11,20]. Adaptation to new hosts species requires mutations and/or recombination events that permit sustained infection within a new host species [11]. Evidence indicates that each of the seven human coronaviruses originated in bats or rodents, with palm civets and camels acting as intermediate hosts for SARS-CoV and MERS-CoV, respectively [11,22,23,24,25,26]. SARS-CoV-2 was found to be most closely related genetically to a horseshoe bat coronavirus called CoV-RaTG13 that was isolated in Yunnan province, China, with 96.2% nucleotide identity between the two viruses [1]. An intermediate host for SARS-CoV-2 has not been conclusively identified, although pangolins have emerged as a potential culprit [27,28,29]. Interestingly, SARS-CoV-2 contains a spike protein receptor binding domain (RBD) and polybasic furin cleavage site that are distinct from CoV-RaTG13; the RBD is directly involved in its cellular entry mechanism through binding to the human angiotensin-converting enzyme 2 (hACE2) receptor, followed by cleavage at the furin cleavage site by the TMPRRS2 transmembrane serine protease [1,30,31]. SARS-CoV-2 is therefore well adapted to infect humans based on the compatibility of the viral spike glycoprotein with the hACE2 receptor, which is abundantly expressed in the human respiratory tract [32].

**Figure 1:**
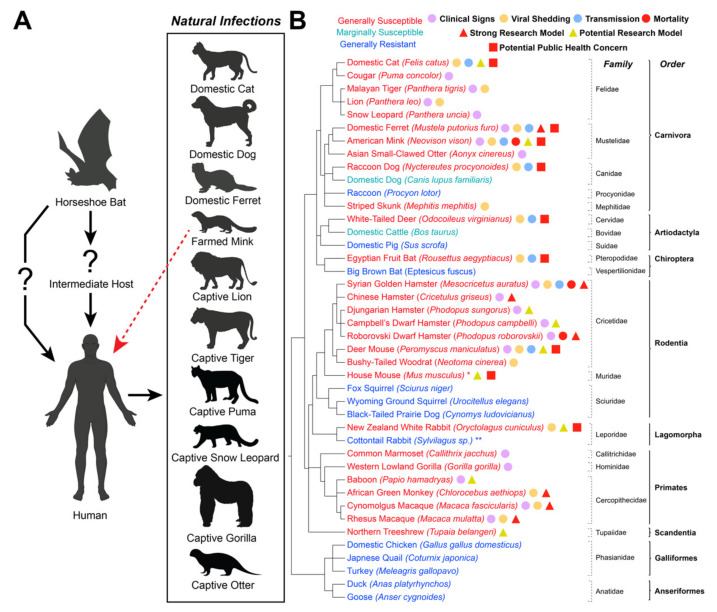
SARS-CoV-2 in Domestic and Wild Animals. (**A**) Disease ecology of SARS-CoV-2. Available evidence suggests that SARS-CoV-2 originated in a horseshoe bat and was then either transmitted directly to humans or through an unidentified intermediate host. Species with documented human-to-animal natural SARS-CoV-2 infections (reverse zoonosis events) are listed, as is the documented mink-to-human transmission of SARS-CoV-2 (red arrow). Created with BioRender.com. (**B**) List of documented species that have either been experimentally or naturally infected with SARS-CoV-2 as of August 2021. Species that are generally susceptible to SARS-CoV-2 are listed in red, marginally susceptible species are listed in cyan, and generally resistant species are listed in blue. * Experimental evidence showed that wild-type mice (*Mus musculus*) are susceptible to mouse-adapted SARS-CoV-2 isolates or certain variants of concern (VOC) that contain an N501Y substitution in the spike protein [138,139,140,141] In contrast, gene-edited mice expressing the human angiotensin-converting enzyme 2 (hACE2) receptor are highly susceptible to infection with ancestral and variant SARS-CoV-2 strains [142,143,144,145,146,147,148,149]. ** Cottontail rabbits (*Sylvilagus* sp.) were resistant to infection [83], although the dose administered was lower than that required for infection of New Zealand white rabbits [82]; therefore, their true susceptibility is unknown. Clear evidence of outwardly observable clinical signs (violet circle), shedding of infectious virus (orange circle), animal-to-animal transmission (blue circle), and mortality (red circle) upon infection is shown for each species. Species considered strong (red triangle) or potential (yellow triangle) research models are noted, as well as species with a potential public health concern (red square). Phylogenetic tree was produced using the phyloT v2 server (https://phylot.biobyte.de, accessed on 26 June 2021) based on the NCBI taxonomy database.

The widespread sustained infection of human populations with SARS-CoV-2 presents the legitimate possibility of reverse zoonotic spillover events, whereby SARS-CoV-2-positive humans infect domestic or wild animals, potentially resulting in the establishment of new reservoir hosts [33,34,35]. Moreover, sustained SARS-CoV-2 infection of animal populations could result in genetic adaptation of the SARS-CoV-2 genome as the virus adapts to a new host. To shed light on the potential for reverse zoonotic events, researchers have been investigating the susceptibility of different animal species to SARS-CoV-2 since the beginning of the pandemic. To date, over thirty different domestic, laboratory, and wild animal species have been subjected to experimental infection with SARS-CoV-2. In addition, various surveillance studies have documented cases of natural SARS-CoV-2 infections in species in contact with human carriers of SARS-CoV-2. In addition to identifying species that could become reservoir hosts, experimental infection studies are also instrumental for establishing pre-clinical animal models that consistently recapitulate COVID-19 disease manifestations for the development and efficacy testing of novel vaccines and therapeutics to combat the disease. In this review, we outline the current knowledgebase surrounding the susceptibility of animal species to both natural and experimental SARS-CoV-2 infection, with an emphasis on domestic and wild animal species at the human–animal interface. A table outlining the disease manifestations of SARS-CoV-2 infection in different domestic and wild species (Table 1) and a figure outlining natural human-to-animal infections, susceptibility/disease severity of different animal species, threats to public health among animal species, and suitability of different susceptible species as pre-clinical models (Figure 1) are included.

**Table 1:** Outline of SARS-CoV-2 susceptibility and disease course in different animal species.

Species	Dose Ranges	Inoculation Route	Infectious Viral Shedding	Clinical Signs	Histopathological Changes	Infectious Virus in Tissues	Transmission	Neutralizing Antibody Response	Susceptible to Re-Infection	Natural Infection	References (Experimental Infections)	References (Natural Infections)
**Domestic cat *(Felis catus)***	10^5^–7 × 10^5^ pfu	Nasal, oral, tracheal, ocular	1–6 DPC (nasal/oral)	Subclinical in most studies (adult/subadult); behavior changes, diarrhea, weight loss in one study; potential severe clinical signs in juveniles	Mild/moderate respiratory tract lesions (adult/subadult), severe in juveniles	Consistent in nasal turbinate, soft palate, trachea, tonsil; isolated detection in lung and intestine	Yes, via direct contact; indirect (aerosol) transmission less effective.	Yes, by 7 DPC	Resistant or limited re-infection	Yes, natural infection in domestic cats and large cats from zoos	[36,37,38,39,40,41,42]	[43,44,45,46,47,48,49,50,51,52,53,54,55,56,57,58,59,60,61,62,63,64,65,66,67]
**Domestic dog (*Canis familiaris)***	10^5^ pfu	Nasal	None	Subclinical	Not reported	Not reported	No transmission	Yes, by 14 DPC	Not tested	Yes	[36,37]	[43,49,50,56,57,59,61,62,68,69,70]
**Syrian golden hamster (*Mesocricetus auratus)***	10^0^–10^5^ pfu	nasal	2–5 DPC (nasal)	Weight loss, lethargy, ruffled fur, hunched posture, respiratory signs, fatal disease reported in older hamsters	Moderate-to-severe lesions in respiratory tract; lesions noted in other tissues	Consistent in nasal turbinate, trachea, lung; 1 sample in brain	Yes, via direct and indirect (aerosol) contact; less efficient via fomites	Yes, by 7 DPC	Resistant to re-infection	None reported	[71,72,73,74,75,76,77,78,79]	
**Chinese hamster (*Cricetulus griseus*)**	10^5^ pfu	Nasal	Not reported (vRNA in oral swabs 2–5 DPC)	Weight loss	Moderate lesions in lungs reported	Detected in lungs at 2–5 DPC	Not tested	Not tested	Not tested	None reported	[80]	
**Djungarian dwarf hamster (*Phodopus sungorus*)**	10^5^ pfu	Nasal	Not reported (vRNA in oral swabs 2–5 DPC)	Subclinical	Moderate-to-severe lesions in lungs reported	Detected in lungs at 2–5 DPC	Not tested	Not tested	Not tested	None reported	[81]	
**Campbell’s dwarf hamster (*Phodopus campbelli*)**	10^5^ pfu	Nasal	Not reported (vRNA in oral swabs 2–5 DPC)	Subclinical	Moderate-to-severe lesions in lungs reported	Detected in lungs at 2–5 DPC	Not tested	Not tested	Not tested	None reported	[81]	
**Roborovski dwarf hamster (*Phodopus roborovskii*)**	5 × 10^4^–10^5^ pfu	Nasal	Not reported (vRNA in oral swabs 2–3 DPC)	Decreased body temperature, severe weight loss, dyspnea, ruffled fur, depressed behavior, required euthanasia between 3–5 DPC	Severe lesions in the lungs reported	Detected in lungs at 2–3 DPC before euthanasia	Not tested	Not tested	Not tested	None reported	[81]	
**New Zealand white rabbit (*Oryctolagus cuniculus*)**	10^4^–10^6^ pfu	Nasal	1–7 DPC (nasal); 1 DPC (oral)	Subclinical	Mild-to-moderate lesions in respiratory tract	Not reported	Not tested	Yes, by 21 DPC	Not tested	None reported	[82]	
**Cottontail rabbit (*Sylvilagus* sp.)**	3 × 10^4^–8 × 10^4^ pfu	Nasal	None	None	None	None	Not tested	None	Not tested	None reported	[83]	
**Domestic ferret (*Mustela putorius furo*)**	5 × 10^2^–5 × 10^6^ pfu	Nasal	2–8 DPC (nasal); 1–5 DPC (oral); 2–4 DPC (saliva); 4 DPC (urine/feces)	Most subclinical; isolated increased body temperature, reduced activity, respiratory signs, reduced activity/appetite, ruffled fur	Mild-to-moderate lesions in respiratory tract	Detected 2–8 DPC in nasal turbinate (high), trachea, larynx, esophagus, soft palate, lung, tonsil	Yes, via direct contact; indirect (aerosol) transmission less effective	Yes, by 10–13 DPC	Resistant to reinfection except with low neutralizing antibodies	Yes	[37,76,84,85,86,87,88,89,90]	[43,91,92]
**American mink (*Neovison vison*)**	5 × 10^6^ pfu	Nasal	2–8 DPC (nasal)	Weight loss, some nasal discharge (experimental); Many asymptomatic; nasal discharge, respiratory distress, reduced activity/feed intake, mortality (natural)	Mild-to-severe lesions in respiratory tract	Detected 4 DPC in nasal turbinate, soft palate, tonsil, lung	Yes, via indirect (aerosol) transmission	Yes, by 18 DPC	Not tested, unlikely based on natural infection data	Widespread infection in farms; natural infection from mink to humans, cats, and dogs	[93]	[43,94,95,96,97,98,99]
**Raccoon dog (*Nyctereutes procyonoides*)**	10^5^ TCID_50_	Nasal	2–4 DPC (nasal/oral)	Most subclinical; isolated lethargy observed	Mild lesions in nasal conchae	None detected	Yes, via indirect (aerosol) transmission	Yes, in some animals by 18 DPC	Not tested	None reported	[100]	
**Domestic cattle (*Bos taurus*)**	10^5^– 3 × 10^7^ TCID_50_	Nasal, tracheal, venous	None (limited vRNA in nasal swabs 2–10 DPC)	Most subclinical; some increased temperature and coughing in calves.	None	None (vRNA detected in one lymph node sample on 10 DPC)	No transmission	Low or absent at 21 DPC	Not tested	None reported	[101,102]	
**Domestic pig (*Sus scrofa*)**	10^5^–2.5 × 10^7^ pfu	Nasal, oral, tracheal, muscular, venous	Not reported (limited vRNA, mostly between 1 and 3 DPC (oral/nasal)	Most subclinical; one study showed isolated ocular nasal discharge, mild depression, cough	None	Detected in only one lymph node 13 DPC	No transmission	Yes, by muscular or venous administration by 22 DPC	Not tested	None reported	[37,84,103,104,105,106]	
**Domestic chicken (*Gallus gallus domesticus*)**	7 × 10^4^–10^6^ pfu	Nasal, choanal, oral, ocular	None	Subclinical	None	None	No transmission	None	Not tested	None reported	[37,84,107,108]	
**Japanese quail (*Coturnix japonica*)**	3 × 10^5^ TCID_50_	Choanal	None	Subclinical	None	Not tested	Not tested	None	Not tested	None reported	[107]	
**Turkey (*Meleagris gallopavo*)**	2 × 10^5^–10^6^ pfu	Nasal, choanal, oral, ocular	None	Subclinical	None	None	Not tested	None	Not tested	None reported	[107]	
**Duck (*Anas platyrhinchos domesticus*)**	10^5^–10^6^ TCID_50_	Nasal, choanal	None	Subclinical	None	Not tested	No transmission	None	Not tested	None reported	[37,107]	
**Goose (*Anser cygnoides*)**	10^6^ TCID_50_	Choanal	None	Subclinical	None	Not tested	Not tested	None	Not tested	None reported	[107]	
**Deer mouse (*Peromyscus maniculatus*)**	2 × 10^4^–10^6^ TCID_50_	Nasal	1–4 DPC (oral); 2–8 DPC (rectal)	Most subclinical; isolated ruffled fur, one study showed weight loss during acute infection	Mild-to-Moderate lesions in respiratory tract, lesions in olfactory epithelium/brain	Detected 2–6 DPC (nasal turbinate, trachea, lung); low in intestine 2–4 DPC	Yes, transmission over two passages	Yes, by 14 DPC	Not tested	None reported	[83,109,110]	
**Bushy-tailed woodrat (*Neotoma cinerea*).**	3 × 10^4^–8 × 10^4^ TCID_50_	Nasal	1–5 DPC (oral)	Subclinical	Mild lesions in lung	3 DPC (nasal turbinate, trachea, lung)	Not tested	Yes, by 28 DPC	Not tested	None reported	[83]	
**Wild House mouse (*Mus musculus)***	3 × 10^4^–8 × 10^4^ TCID_50_	Nasal	None	Subclinical	None	None	Not tested	None	Not tested	None reported	[83]	
**Fox squirrel (*Sciurus niger*)**	3 × 10^4^–8 × 10^4^ TCID_50_	Nasal	None	Subclinical	None	None	Not tested	Not tested	Not tested	None reported	[83]	
**Wyoming ground squirrel (*Urocitellus elegans*)**	3 × 10^4^–8 × 10^4^ TCID_50_	Nasal	None	Subclinical	None	None	Not tested	Not tested	Not tested	None reported	[83]	
**Black-tailed prairie dog (*Cynomys ludovicianus*)**	3 × 10^4^–8 × 10^4^ TCID_50_	Nasal	None	Subclinical	None	None	Not tested	None	Not tested	None reported	[83]	
**Asian small-clawed otter (*Aonyx cinereus*)**	Not applicable	Not tested	Not tested	Respiratory signs, lethargy	Not tested	Not tested	Yes, via direct contact	Not tested	Not tested	Yes		[111,112]
**Striped skunk (*Mephitis mephitis*)**	3 × 10^4^–8 × 10^4^ TCID_50_	Nasal	2–7 DPC (nasal); 2–5 DPC (oral)	Subclinical	None	3 DPC (nasal turbinate)	Not tested	Yes, by 28 DPC	Not tested	None reported	[83]	
**Raccoon (*Procylon lotor*)**	3 × 10^4^–8 × 10^4^ TCID_50_	Nasal	None	Subclinical	None	None	Not tested	None	Not tested	None reported	[83]	
**White-tailed deer (*Odocoileus virginianus*)**	10^6^–10^7^ TCID_50_	Nasal	1–5 DPC (nasal); 3 DPC (oral); 5 DPC (rectal); 1 DPC (feces)	Most subclinical; elevated body temperature; some ocular/nasal discharge	Mild lesions in respiratory tract	4 DPC (trachea/bronchi)	Yes, via direction and indirect (aerosol) contact	Yes, by 7 DPC	Not tested	Yes, determined via serology and RT-qPCR	[113,114]	[115,116]
**Northern Tree shrew (*Tupaia belangeri*)**	10^6^–10^7^ TCID_50_	Nasal, oral, ocular	None reported; no vRNA shedding in one study; variable vRNA shedding in one study	Most subclinical; increase in body temperature	Mild-to-moderate lesions in lungs; several histopathological changes reported in non-respiratory tissues	4–7 DPC (trachea, lung, pancreas)	Not tested	Reportedly yes, unknown DPC	Not tested	None reported	[117,118]	
**Egyptian fruit bat (*Rousettus aegyptiacus*)**	10^5^ TCID_50_	Nasal	2 DPC (one oral swab); (vRNA 2–12 DPC (oral); 2–4 DPC (feces)	Subclinical	Mild-to-moderate lesions in upper respiratory tract; some mild lesions in lung	4 DPC (trachea, lung)	Yes, to proportion of bats via direct contact	Yes, weak response by 8 DPC	Not tested	None reported	[84]	
**Big brown bat (*Eptesicus fuscus*)**	10^5^ TCID_5_	Nasal, Oral	None	Subclinical	None	None	None	None	Not tested	None reported	[119]	
**Rhesus macaque (*Macaca mulatta*)**	10^4^–5 × 10^6^ pfu	Nasal, oral, tracheal, ocular, venous	1–5 DPC (nasal); 1–6 DPC (oral); 9 DPC (rectal)	Subclinical, or elevated body temperature, decreased activity, appetite, body weight; changes in respiratory pattern	Mild-to-moderate lesions in respiratory tract	3 DPC (lungs)	Not tested	Yes, by 8 DPC	Resistant to reinfection	None reported	[120,121,122,123,124,125,126,127,128,129,130,131,132]	
**Cynomolgus macaque (*Macaca fascicularis*)**	10^6^–2 × 10^7^ pfu	Nasal, oral, tracheal, ocular, venous	1–7 DPC (nasal, oral); 1–3 DPC (conjunctival)	Subclinical, or elevated body temperature, decreased appetite and body weight.	Mild lesions in respiratory tract	3 DPC (lungs)	Not tested	Yes, by 7 DPC	Not tested	None reported	[122,128,131,133]	
**African green monkey (*Chlorocebus aethiops*)**	1.5 × 10^3^–2.5 × 10^6^ pfu	Nasal, oral, tracheal, ocular, aerosol	2–7, 21 DPC (nasal); 2–9, 21 DPC (oral); 2–5, 14 DPC (rectal)	Decreased appetite, anorexia, elevated body temperature, changes in respiratory rate; ARDS in two animals	Mild respiratory lesions; severe in two animals with ARDS	5 DPC (lungs)	Not tested	Yes, by 5 DPC	Resistant to reinfection	None reported	[132,134,135,136]	
**Baboon (*Papio hamadryas*)**	10^6^ pfu	Nasal, tracheal, ocular	Not reported; vRNA detected 3–17 DPC (nasal/rectal)	None reported	Moderate respiratory tract lesions	Not reported; vRNA detected in lungs at 14/17 DPC	Not tested	Not reported	Not tested	None reported	[127]	
**Common marmoset (*Callithrix jacchus*)**	10^6^ pfu	Nasal, tracheal, ocular	Not reported; vRNA detected 2–12 DPC (nasal), 2–10 DPC (oral/rectal), 2–8 DPC (blood), 6–21 DPC (feces).	Most subclinical, increased body temperature.	Mild respiratory tract lesions.	Not reported; vRNA detected in lungs at 3/14 DPC	Not tested	None detected	Not tested	None reported	[122,127]	
**Western lowland gorilla (*Gorilla gorilla*)**	Not applicable	Not tested	Not tested	Respiratory signs	Not tested	Not tested	Yes, via direct contact	Not tested	Not tested	Yes		[137]

## 2. Main Text

### 2.1. Domestic Animals

#### 2.1.1. Cats

Domestic cats (*Felis catus*) have been shown by multiple investigators to be highly susceptible to both experimental and natural SARS-CoV-2 infection. Experimentally infected cats generally exhibit an asymptomatic and self-limited course of disease, primarily localized to the upper respiratory tract. Cats also readily transmit the virus to naive cats in close contact under experimental conditions. In addition, cats mount a robust neutralizing immune response that appears to protect them from re-infection, at least in the short term.

Experimental studies investigating SARS-CoV-2 susceptibility in cats have used a 1 × 10^5^ to 7 × 10^5^ pfu viral dose (doses measured in TCID_50_ were converted to pfu by multiplying by 0.7, or pfu to TCID_50_ by dividing by 0.7, throughout the review for simplicity) with either nasal [36,37,38], nasal/oral [39], or nasal/oral/tracheal/ocular [40,41] administration. Most experiments used subadult 3- to 18-month-old cats, although juvenile (1- to 3-month-old) [37] and adult (5- to 8-year-old) [36] cats were also analyzed.

All experimental inoculations in cats resulted in a productive SARS-CoV-2 infection [36,37,38,39,40,41]. Viable virus was isolated from nasal and oropharyngeal swabs as early as 1 day post challenge (DPC) with viral shedding continuing up to 6 DPC [36,40]. Viral RNA was detected beyond this period in both oropharyngeal and nasal swabs from 1 to 10 DPC and rectal swabs from 3 to 14 DPC [38,39], although no viable virus was recovered from rectal swabs in a separate study [40]. Viral RNA was also detected in fecal samples, but not urine samples [37,39].

Despite clear evidence of SARS-CoV-2 infection, sub-adult and adult cats in most studies did not exhibit any clinical signs such as increased body temperature, weight loss, respiratory distress, conjunctivitis, or change in behavior [36,37,39,40]. One recent study did observe clinical signs in sub-adult cats aged 8 to 18 months old, reporting arching of the back, weight loss <10%, and diarrhea [38]. Despite the absence of clinical signs in most studies, mild-to-moderate histopathological changes were consistently observed in the upper and lower respiratory tracts, with nasal turbinate, trachea, and lungs all exhibiting pathology associated with viral infection [36,38,39,41]. These pathological changes generally began to resolve after the acute infection period, although two studies demonstrated that histopathological changes persisted a month after resolution of the acute infection [36,41]. The gastrointestinal, cardiovascular, and nervous systems did not show any gross or histological lesions, nor did any other major organs or lymphoid tissues [36,39]. Interestingly, one study did find that juvenile cats (aged 70 to 100 days) that either died or were humanely euthanized at 3 DPC exhibited severe lesions in the upper and lower respiratory tracts and histological lesions in the small intestine [37]. However, whether these cats exhibited overt clinical signs and whether they were euthanized or died due to SARS-CoV-2 infection was not clearly stated [37]. Moreover, only slightly older cats in separate studies did not exhibit any clinical signs [40,41]. Therefore, it is possible that juvenile cats are more susceptible to SARS-CoV-2 infection and severe COVID-19 disease than sub-adult/adult cats. Together, these studies indicate that SARS-CoV-2 generally causes a subclinical infection accompanied by generally mild-to-moderate pathological changes in the respiratory tract of domestic cats.

Consistent with histopathological findings, infectious virus was consistently detected in nasal turbinate, soft palate, tonsil, and trachea tissues during the shedding period, but was not recovered from the lungs of subadult/adult cats, except in isolated animals at 3 DPC, indicating primary localization of viral replication to the upper respiratory tract [36,37,41]. Interestingly, viable virus was recovered from the lungs of juvenile cats at 3 and 6 DPC and the small intestine of a juvenile cat at 3 DPC, further suggesting a higher susceptibility in this population [37]. Viral RNA was widely detected in other tissues, including the tracheobronchial lymph node, mesenteric lymph node, spleen, olfactory bulb, liver, heart, and kidney, suggesting systemic distribution of the virus [39].

Several experimental studies also clearly demonstrated that cat-to-cat SARS-CoV-2 transmission occurs via both direct contact [36,38,39,40] and indirect contact via aerosols [37]. Infectious virus was detected in clinical samples from naïve cats co-housed with inoculated cats one or two days after exposure, with a similar disease course compared to inoculated cats [36,40]. Indirect aerosol transmission was demonstrated in only a proportion of cats exposed by this method, suggesting a lower efficiency compared to direct contact [37]. Interestingly, one study indicated that four serial transmissions of SARS-CoV-2 in cats results in an attenuation of viral shedding and histopathological symptoms in cats, in contrast to sustained human-to-human transmission [38]. The basis of the attenuation after passage in cats is not clear but is of significant interest and will require further investigation.

The experimental studies also demonstrate that cats mount a strong immune response against SARS-CoV-2, with neutralizing antibodies detected as early as 7 DPC [36,39]. Moreover, re-inoculation of cats at 21 or 28 DPC did not result in productive re-infection and the neutralizing antibody response increased following re-infection, suggesting an anamnestic immune response [36,41,42]. Furthermore, re-infected cats did not transmit SARS-CoV-2 to naïve cats in direct contact [42].

Natural, human-to-cat SARS-CoV-2 infection in domestic and captive cats has been demonstrated in Asia, Europe, and the Americas [43]. Several instances of cats testing positive for SARS-CoV-2 via RT-qPCR have been documented in households with SARS-CoV-2 infected individuals [44,45,46,47,48,49,50,51,52,53,54]. In addition, surveillance studies have detected antibodies against SARS-CoV-2 in cats which lived in close contact with infected humans or stray cats in areas of active outbreaks [49,50,55,56,57,58,59,60,61,62,63,64,65]. Moreover, big cats (tigers, lions, cougars, and a snow leopard) have tested positive for SARS-CoV-2 after exposures to SARS-CoV-2 infected zookeepers [43,66,67]. Interestingly, some of these large cats exhibited mild respiratory signs not observed in the experimental infections with domestic cats [43,67]. Likewise, several naturally infected domestic cats exhibited clinical signs (respiratory signs, nasal/ocular discharge, and loss of appetite) that may be attributed to comorbidities not represented in animals enrolled in experimental studies [45,52,53]. These data clearly demonstrate that both domestic and big, captive cats are susceptible to natural human-to-cat transmission.

Despite the documented cases of natural SARS-CoV-2 infections in cats, the low prevalence of natural infection suggests that human-to-cat transmission may be somewhat inefficient. Among published surveillance studies, the highest documented seroconversion prevalence in cats was 23% in a population in close contact with SARS-CoV-2-infected owners during an active outbreak [62]. Moreover, several surveillance studies of cat populations during active outbreaks have failed to detect SARS-CoV-2 or identify any seroconversion among populations with exposure to the virus [150,151,152,153]. A particularly interesting study demonstrated that a group of SARS-CoV-2 infected students did not infect any of the nine cats that were in close prolonged contact with them [154]. Considering SARS-CoV-2 has infected over 200 million individuals worldwide, the evidence of natural infection of cats seems lower than would be expected if the virus readily transmits from humans to cats; however, the absence of clinical signs and the generally low rate of testing does question the accuracy of these data. Therefore, the possibility that SARS-CoV-2 becomes endemic in either domestic or feral cat populations is still unclear but appears unlikely. However, precautions should be taken to avoid SARS-CoV-2 transmission between cats, humans, and other susceptible hosts in close contact, especially those within the same household or in veterinary clinics, shelters, and catteries.

Overall, cats are clearly susceptible to SARS-CoV-2 infection, although the infection is generally free of clinical signs with only mild-to-moderate pathological changes and a relatively short duration of transmission. Natural infection of cats has been clearly demonstrated, but human-to-cat transmission seems substantially lower than human-to-human infection and widespread transmission within cat populations is unlikely based on current data. Cats could indeed make a good pre-clinical model to understand SARS-CoV-2 pathogenesis in greater detail and to develop therapeutics and vaccines. However, the absence of overt clinical signs, inherent difficulties in handling cats under high biocontainment conditions required for SARS-CoV-2, high cost per animal, and ethical considerations regarding companion animals in research makes their use as an experimental model somewhat limited.

#### 2.1.2. Dogs

Domestic dogs (*Canis familiaris*) have been shown in several different studies to have a low susceptibility to SARS-CoV-2 via experimental and natural infection. There is evidence of limited viral replication in a proportion of infected dogs, but no evidence of prolonged acute infection necessary for sustained transmission. Interestingly, most experimentally infected dogs develop an antibody response against SARS-CoV-2, and seroconversion has also been documented in natural human-to-dog transmission events.

Experimental studies of SARS-CoV-2 susceptibility in dogs were performed using a 10^5^ pfu viral dose administered intranasally in research-bred beagles [36,37]. One of the experiments used juvenile (3-month-old) dogs [37], and the other one used adults (5 to 6 years old) [36].

Both studies demonstrated limited SARS-CoV-2 replication in dogs after nasal inoculation. Viable virus was not isolated from any swab or tissue sample for the duration of either study, which was 14 or 42 days [36,37]. SARS-CoV-2 RNA was detected in rectal swabs only in two out of five inoculated dogs on 2 DPC and in one of these dogs at 6 DPC, suggesting that some level of viral replication occurred in a few experimentally inoculated animals [37]. No viral RNA was detected in any other swabs or tissues, such as lung, trachea, nasal turbinate, or tonsil, collected throughout the studies.

None of the experimentally inoculated dogs exhibited any clinical signs throughout the 14- to 42-day observation period of the respective studies [36,37]. Gross and histopathological findings were not reported, presumably due to the absence of any appreciable abnormalities. Importantly, experimentally infected dogs were unable to transmit the virus to naïve co-housed animals, with no detection of viral RNA in contact animals and no evidence of seroconversion [37].

Despite the limited evidence of viral replication and absence of clinical signs, some infected dogs did mount an immune response against SARS-CoV-2. In one study, half of the inoculated dogs (2/4) developed antibodies against SARS-CoV-2 by 14 DPC [37]. In the other study, all dogs (3/3) developed neutralizing antibodies starting at 14 DPC that peaked at 21 DPC [36]. These results demonstrate that dogs have a low susceptibility to experimental SARS-CoV-2 infection and can generate an antibody response against the virus.

Surprisingly, natural infection of dogs with SARS-CoV-2 has been clearly demonstrated in cases in Asia, Europe, and the Americas [43,68]. Two dogs from Hong Kong tested positive for SARS-CoV-2; a 17-year-old dog with comorbidities tested positive over a 13-day period, and a 2-year-old dog that tested positive and showed shedding of viable virus in oral and nasal swabs [68]. Multiple surveillance studies also demonstrated a low prevalence of seropositive dogs in regions with active SARS-CoV-2 outbreaks [49,50,56,57,59,61,62,69,70]. However, other surveillance studies failed to show any transmission or seroconversion in dogs, including the study involving SARS-CoV-2-infected students in prolonged contact with several pet dogs [44,154]. Moreover, the 2-year-old dog that shed viable virus in Hong Kong failed to infect another dog in the same household [68]. These data indicate that dogs are susceptible to SARS-CoV-2 via natural infection, although no clear evidence suggests that they are likely to become reservoir hosts or have the potential to infect humans or even other dogs in close contact. Interestingly, the isolation of live virus and evidence of extended shedding of viral RNA reported in naturally infected dogs was not observed after experimental infection of research-bred beagles [36,37]. This suggests that other factors such as age, breed, and co-morbidities may influence the susceptibility of dogs to productive SARS-CoV-2 infection.

Cumulatively, dogs have been shown to have low susceptibility to natural and experimental SARS-CoV-2 infections, with an absence of clinical signs and limited viral replication, but with clear evidence of seroconversion. The likelihood that dogs become a reservoir species is rather low, especially considering the lack of transmission observed between dogs. Dogs are unlikely to be useful pre-clinical models for SARS-CoV-2 research.

#### 2.1.3. Hamsters

Several different species of hamsters are highly susceptible to SARS-CoV-2 by experimental infection and can transmit the virus to naïve hamsters, although there is currently no evidence of natural human-to-hamster infections [71,72,73,74,75,76,77,78,80,81]. Syrian golden hamsters (*Mesocricetus auratus*) have been studied most extensively, and exhibit acute disease characterized by mild-to-moderate clinical signs with moderate-to-severe pathological changes in the respiratory tract [71,72,73,74,75,76,77,78]. Chinese hamsters (*Cricetulus griseus*), Campbell’s dwarf hamster (*Phodopus campbelli*), and Djungarian hamster (*Phodopus sungorus*) have also been investigated and exhibit a similar susceptibility and disease progression after SARS-CoV-2 infection compared to Syrian hamsters [80,81]. Interestingly, Roborovski dwarf hamsters (*Phodopus roborovskii*) develop an acute, terminal disease with severe clinical signs; they therefore have a disease progression comparable to fatal human COVID-19 [81]. Hamsters have therefore emerged as the most promising pre-clinical animal model for SARS-CoV-2 infection.

Syrian golden hamsters have been the most widely used species for experimental SARS-CoV-2 studies. Most studies inoculated 4- to 34-week-old hamsters with 10^3^ to 10^5^ pfu intranasally [71,72,73,74,76,78,79]. One study inoculated older Syrian golden hamsters (10 to 20 months old) with 7 × 10^4^ pfu SARS-CoV-2 [77]. Two additional studies used a wide range of doses from 10^0^ to 10^5^ 50% tissue culture infectious dose (TCID_50_) to determine the 50% infectious dose (ID_50_) [75,77].

All experimental infections resulted in a productive SARS-CoV-2 infection in Syrian golden hamsters [71,72,73,74,75,76,77,78,79]. Infected hamsters shed infectious virus in nasal washes from 2 to 5 DPC during the approximate period of acute infection [74]. Viral RNA was detected in nasal washes continuously for 14 days, and viral RNA was also detected in oral and rectal swabs up to 10 DPC [71,74,75,76,77,78,79]. Viral RNA was not found in urine in one study [76]. The ID_50_ required to cause infection was determined to be only 5 TCID_50_ when SARS-CoV-2 was administered intranasally [75].

Acute infection in Syrian golden hamsters was accompanied by obvious clinical signs [71,72,73,74,75,79]. A decrease in body weight between 1 to 6 DPC was consistently observed, and weight was gradually regained by 14 DPC [71,72,73,74,75,76,77,79]. The weight loss correlated with the infectious SARS-CoV-2 dose administered [73,75,76,77]. Hamsters also developed lethargy, ruffled fur, hunched posture, and changes in respiratory function during the period of acute infection, which generally began to improve by 7 DPC [72,75,77]. In two studies, older hamsters (>7 months old) were found to have more severe clinical signs than younger ones [71,77], with a proportion of hamsters over 10 months old inoculated with a 10^5^ TCID_50_ dose resulting in death due to respiratory disease in one of the studies [77].

The period of acute SARS-CoV-2 infection in Syrian golden hamsters was accompanied by moderate-to-severe lesions in nasal turbinates, trachea, and lungs [71,72,73,74,75,76,78,79]. The observed pathology was directly correlated with the infectious dose administered [77]. Respiratory pathology coincided with the presence of infectious virus, which was consistently detected in the nasal turbinates, trachea, and lungs between 2 and 6 DPC [71,72,73,74,75,76]. Histopathological lesion began to improve by 14 DPC with only sporadic detection of infectious virus in respiratory tissues after 7 DPC [71,72,73,74,75,78,79]. Interestingly, a recent study detected higher levels of subgenomic SARS-CoV-2 RNA in the respiratory tract of Syrian golden hamsters infected with the B.1.617 (Delta) variant of concern (VOC) at 14 DPC, which warrants additional investigation into the transmission capacity of this VOC [78]. Among other major organs tested, low levels of viral RNA were detected sporadically during the period of acute infection in the spleen, liver, kidneys, brain, heart, lymph nodes, intestine, adrenal glands, reproductive organs, and blood, indicating systemic distribution of SARS-CoV-2 during acute infection [71,72,74,75,76,77,79]. One detailed study reported histopathological changes in the spleen and intestine [72]; another one reported lesions in the spleen, lymph nodes, kidneys, adrenal glands, and reproductive organs during acute infection with focal lesions persisting in the liver, gallbladder, heart, and lymph nodes beyond 18 DPC [79]. Infectious virus was isolated from brain tissue (including the olfactory bulb) on 3 DPC in one study [73], and a decrease in the number of olfactory cells at the nasal mucosa at 2 DPC was noted in another study [74]. Due to the widespread use of Syrian golden hamsters as a model for COVID-19, and the importance of lung pathology as a marker of disease, standardization of reporting lung pathology has been proposed and should be updated as more detailed information is provided [155].

Several studies investigated the ability of Syrian golden hamsters to transmit the virus to naïve hamsters under various conditions [72,74,77]. Naïve hamsters in direct contact with inoculated hamsters via co-housing at 1 DPC became productively infected with SARS-CoV-2, exhibiting similar patterns of viral shedding and clinical signs [72,74]. Interestingly, introducing naïve hamsters into inoculated hamsters’ cages at 6 DPC did not result in productive infection and no clinical signs were observed, indicating that transmission to contacts does not occur after 6 DPC [74]. A lower dose administered to principal inoculated hamsters (10^4^ TCID_50_) still resulted in effective transmission via direct contact, but without weight loss in the contact hamsters [77]. Aerosol transmission, achieved by housing naïve hamsters in wire cages adjacent to infected hamsters for 8 h on 1 DPC, also resulted in efficient transmission [74]. Lastly, transmission via fomites, achieved by introducing naïve hamsters in previously occupied cages for 48 h, only resulted in a proportion of hamsters becoming infected, suggesting a low efficiency of fomite transmission [74].

Neutralizing antibodies were detected in infected Syrian golden hamsters as early as 7 DPC [71,72,73,74,76,78]. Not surprisingly, re-challenge of recovered hamsters with SARS-CoV-2 resulted in low or no virus replication [73,77], and no transmission via direct contact with re-infected hamsters [77], demonstrating that challenge provides a robust protective immunity against SARS-CoV-2.

A single study also found that Chinese dwarf hamsters are highly susceptible to SARS-CoV-2 [80]. Five- to 7-week-old Chinese dwarf hamsters were infected with 10^5^ pfu intranasally. The only clinical sign observed was a decrease in body weight between 1 and 5 DPC which was not fully regained by 14 DPC. Viral RNA was detected in oral swabs and lung samples between 2 and 5 DPC, with only a small level remaining in the lungs at 14 DPC. Low levels of viral RNA were also detected in the blood of some hamsters between 2 and 5 DPC. The lungs of Chinese hamsters exhibited respiratory pathology comparable to infected Syrian golden hamsters, but with a milder and more prolonged course of pneumonia. Infectious virus was detected in the lungs between 2 and 5 DPC but was not present at 14 DPC. Therefore, Chinese hamsters are clearly susceptible to SARS-CoV-2 with a similar disease progression when compared to Syrian golden hamsters.

Campbell’s dwarf hamsters and Djungarian dwarf hamsters were also shown to be susceptible to SARS-CoV-2 infection but showed somewhat milder clinical signs compared to Chinese dwarf and Syrian golden hamsters [81]. Five- to 7-week-old hamsters were inoculated with a dose of 10^5^ pfu SARS-CoV-2 intranasally. Both hamster species were productively infected, with infectious virus recovered from the lungs between 2 and 5 DPC, but not on 14 DPC. Viral RNA detected in oral swabs followed the same pattern, which is consistent with the acute disease progression seen in Syrian golden hamsters. Interestingly, viral RNA was detected in the blood of some of the animals from both species between 2 and 5 DPC, indicating a systemic infection. Histopathological changes in the lungs of both hamster species were similar to Syrian golden hamsters. Notably, Campbell’s and Djungarian hamsters did not exhibit any significant changes in body temperatures, weight loss, or other clinical signs.

In contrast to the mild symptoms seen in Campbell’s and Djungarian dwarf hamsters, the same study found that 5- to 7-week-old Roborovski dwarf hamsters, inoculated with 10^5^ pfu SARS-CoV-2 intranasally, suffered fulminant terminal disease [81]. Roborovski dwarf hamsters exhibited severe clinical signs, including a decrease in body temperature at 1 DPC, weight loss up to 30% on 3 DPC, dyspnea, sniffling, ruffled fur, and depressed behavior. High viral titers were present in the lungs of Roborovski hamsters from 2 to 3 DPC. High levels of viral RNA were also detected in oral swabs at this time, and viral RNA was also detected in the blood. By 3 DPC, all infected Roborovski hamsters were terminally ill and humanely euthanized. Infection with a lower dose (5 × 10^3^ pfu) of SARS-CoV-2 delayed the clinical signs, but the hamsters nonetheless exhibited signs of terminal disease and were euthanized on 4 or 5 DPC. Roborovski dwarf hamsters infected with the high dose developed highly destructive and diffuse damage throughout the lungs with alveolar epithelial necrosis as early as 2 DPC. Roborovski dwarf hamsters are therefore a species that develops consistent severe, fatal illness upon SARS-CoV-2 infection. Interestingly, the authors determined that there are no differences in SARS-CoV-2 spike-interacting ACE2 residues in Roborovski dwarf hamsters compared to the other dwarf hamsters or the Syrian golden hamster. Therefore, the basis of the severe disease manifestations seen in the Roborovski hamsters will require additional investigation.

To date, there have been no documented cases of natural human-to-hamster transmission of SARS-CoV-2 in pet hamsters. Considering hamsters are generally kept in cages, transmission from a COVID-19-infected person to a pet hamster could be easily avoided by limiting contact with the animals. This should be advised based on the hamsters’ course of disease, particularly with Roborovski dwarf hamsters. The introduction of SARS-CoV-2 into a group of co-housed hamsters would likely spread rapidly. Studies to investigate SARS-CoV-2 adaptation in hamsters are warranted to determine the potential for mutations, which could affect virus virulence.

Hamsters, particularly Syrian golden hamsters, are the most promising pre-clinical model of SARS-CoV-2 due to their consistent clinical signs and ability to transmit the virus to other hamsters, combined with their relative ease of housing and handling in biocontainment facilities. Syrian golden hamsters have already been used extensively to gain insights into the basic science of SARS-CoV-2 pathogenesis [156,157,158,159,160,161] and to test therapeutic interventions [162,163,164,165,166,167] and vaccines [168,169,170,171,172]. Moreover, there is clear evidence that older Syrian golden hamsters and Roborovski hamsters develop respiratory disease and lung pathology that recapitulates the severe disease found in older COVID-19 patients, and therefore could be highly informative models [77,81].

#### 2.1.4. Rabbits

New Zealand white rabbits (*Oryctolagus cuniculus*) have been shown in one study to be susceptible to experimental infection with SARS-CoV-2 [82]. Rabbits developed a subclinical infection, mainly of the upper respiratory tract, associated with an acute period of viral shedding followed by seroconversion. Transmission between rabbits has not been established and there is no evidence of natural infection in rabbits.

Three-month-old New Zealand white rabbits were inoculated with 10^4^, 10^5^ or 10^6^ TCID_50_ SARS-CoV-2 intranasally [82]. Rabbits infected with the 10^6^ TCID_50_ dose demonstrated productive viral infection, with infectious virus recovered from nasal swabs between 1 and 7 DPC. Infectious virus was isolated from oral swabs only on 1 DPC and was not detected in rectal swabs. Viral RNA was detected in nasal swabs until 21 DPC, in oral swabs until 14 DPC, and in rectal swabs until 9 DPC. Inoculation of rabbits with the lower (10^5^ and 10^4^ TCID_50_) doses resulted in productive infection only in the group receiving the 10^5^ TCID_50_ dose, as determined by viral RNA shedding in nasal and oral swabs. Despite productive SARS-CoV-2 infection, all infected rabbits remained asymptomatic throughout the study. Interestingly, no viral RNA was detected in lung tissue in rabbits inoculated with the 10^6^ TCID_50_ dose, but mild-to-moderate histopathological changes in the nasal turbinates, trachea, and lungs were observed at 4 DPC, along with enlargement of the tracheobronchial lymph nodes in some of the animals. All animals infected with the 10^6^ TCID_50_ dose developed neutralizing antibodies at 21 DPC.

Though not a domestic species, a single study did find that three wild-caught cottontail rabbits (*Sylvilagus* sp.) were not susceptible to experimental SARS-CoV-2 infection when inoculated intranasally with a viral dose between 3 × 10^4^ to 8 × 10^4^ TCID_50_ [83]. The cottontail rabbits did not exhibit any viral shedding at 3 DPC, at which point they were euthanized. The reason for the apparent resistance of the *Sylvilagus* cottontail rabbits to SARS-CoV-2 may be explained by the virus challenge dose used in this study, which was lower than the 10^5^ TCID_50_ dose required to elicit productive infection in the related *Oryctolagus* New Zealand rabbits. Additional studies using a higher virus challenge dose should be performed before making concrete predictions regarding SARS-CoV-2 susceptibility in *Sylvilagus* rabbit species.

There are currently no reports regarding natural human-to-rabbit SARS-CoV-2 infections, and their ability to transmit the virus to other rabbits is currently unknown. The inability for rabbits to become infected upon experimental intranasal inoculation with a 10^4^ TCID_50_ dose suggests that a human-to-rabbit transmission event may be inefficient. Regardless, pet owners and farm workers should avoid contact with rabbits if they are infected with SARS-CoV-2. Although the experimental study did not include a transmission component, the pattern of virus shedding, especially from the nasal cavities of rabbits, is reminiscent of several other susceptible animal species that readily transmit the virus. Natural infection in rabbits may be difficult to detect due to the lack of clinical signs and will therefore require active surveillance. Additional studies of SARS-CoV-2 infection in domestic rabbits should be performed to investigate potential genetic adaptations of the virus and to determine the potential for transmission. Rabbits could be used as a pre-clinical model for the development of SARS-CoV-2 countermeasures; however, the absence of clinical signs may limit their usefulness.

#### 2.1.5. Ferrets

Domestic ferrets (*Mustela putorius furo*) are susceptible to both experimental and natural SARS-CoV-2 infection. Experimental inoculation of ferrets with SARS-CoV-2 results in acute infection primarily localized to the upper respiratory tract, accompanied by mild clinical signs in some instances. Ferrets readily transmit the virus to naïve animals via aerosol or direct contact and mount a robust neutralizing antibody response. Therefore, ferrets seem to be a good pre-clinical animal model for SARS-CoV-2 research, including virus transmission studies. Although natural infection of pet ferrets has only been demonstrated twice to date, their high susceptibility to experimental infections warrants surveillance and precautions be taken in domestic and clinical settings.

Most experimental inoculations of SARS-CoV-2 in ferrets have used doses ranging from 1 × 10^5^ to 5 × 10^6^ pfu per animal administered intranasally [37,84,85,86,87,88,89]. Additional studies have investigated the effects of lower doses (5 × 10^2^ to 5 × 10^4^ pfu) that were also administered intranasally [76,88,90]. Relatively young ferrets were used in all studies so far, ranging from 3 to 24 months old [37,76,84,85,86,87,88,90].

All experimental inoculations of ferrets resulted in a productive SARS-CoV-2 infection [37,76,84,85,86,87,88,89,90]. Viable virus was detected in nasal washes/swabs between 2 and 8 DPC [37,76,84,85,86,87,88,89,90], oral swabs between 1 and 5 DPC [85,89], and saliva between 2 and 4 DPC [86]. Viable virus was not recovered from rectal swabs in multiple studies [37,76,85], but one study determined that clarified urine and fecal samples from 4 DPC could productively infect naïve ferrets, thus demonstrating the presence of viable virus [86]. Detection of viral RNA in nasal washes/swabs and rectal swabs generally followed a similar pattern compared to infectious virus, with viral RNA detected between 2 and 8 DPC in most studies [37,76,84,86,87,90]. However, other studies reported an extended detection of viral RNA in nasal, oral, and/or rectal swabs up to 15 or 19 DPC [76,85,88]. In contrast, virus shedding was found to be significantly reduced in clinical samples in ferrets administered lower doses of SARS-CoV-2 [88,90]. Viral RNA was detected in saliva, urine, and feces between 2 and 8 DPC in one study [86] and on the fur of infected ferrets in another study at a site of mutual grooming [87]. These results indicate that ferrets become productively infected with SARS-CoV-2 upon experimental inoculation and efficiently shed viable virus from the nasal, oral, and rectal cavities for a period of up to eight days.

Several experimental SARS-CoV-2 infection studies with ferrets reported a completely subclinical infection [84,85,89,90]. However, others reported mild clinical signs after infection, including increased body temperature, reduced activity, occasional coughing, snoring, reduced appetite, and ruffled fur [37,76,86,87,88]. None of the studies noticed reduced body weight or gastrointestinal tract issues [37,76,84,85,86,87,88,90].

Despite the absence of clinical illness, mild-to-moderate histopathological changes were observed in the upper and lower respiratory tract, with lesions detected in the nasal turbinate and lung that improved by the end of the study [37,76,84,86,87,88,90]. The presence of infectious SARS-CoV-2 virus isolated from ferret tissues varied between studies, but infectious virus was consistently detected in nasal turbinate, trachea, larynx, esophagus, soft palate, lung, and tonsil samples between 2 and 8 DPC [37,76,84,86,87]. The presence of viral RNA in tissues also varied, but was primarily detected between 4 and 8 DPC in the nasal turbinate, trachea, larynx, esophagus, soft palate, lung, tonsil, brain, skin, muscle, tongue, stomach, intestine, kidney, serum and tonsil, indicating a localization to the respiratory tract with some systemic involvement [37,76,84,86,87,88]. Histopathological changes and SARS-CoV-2 detection in tissues significantly reduced or were absent in ferrets administered lower viral doses (5 × 10^2^ pfu) [88]. Several studies clearly demonstrated that the highest level of both infectious virus and viral RNA was recovered from the nasal turbinates, indicating a primary localization of viral replication in the upper respiratory tract [76,86,87,88].

Several studies investigated the ability of ferrets to infect naïve sentinel ferrets via direct or indirect (aerosol) contact [84,85,86]. All naïve ferrets in direct contact with inoculated ferrets developed productive infection with comparable disease course, clinical signs, and pathology [84,86]. Conversely, only a portion of the ferrets subjected to indirect/aerosol exposure became infected, indicating aerosol transmission is less efficient than direct contact [85,86,89].

Two studies determined that inoculated ferrets developed a neutralizing antibody response by 10 to 13 DPC [37,76,86,88], although an additional study did not detect neutralizing titers until 21 DPC in infected ferrets [84]. Ferrets receiving a low (5 × 10^2^ pfu) dose of SARS-CoV-2 had comparatively low neutralizing antibody titers [88]. One study determined that a proportion of ferrets became successfully re-infected after primary SARS-CoV-2 infection and recovery, although the neutralizing antibody titers present in ferrets prior to re-infection were absent or low [87]. Another recent study determined that ferrets with low neutralizing antibody titers could indeed become productively re-infected with SARS-CoV-2 and were capable of transmitting virus to ferrets in direct contact, which might have implications for SARS-COV-2 transmission in humans [90].

An intriguing aspect of SARS-CoV-2 infection of ferrets is the documented emergence of non-synonymous mutations in ORF1ab and the spike gene after experimental infection [84,85,87]. Of particular interest are three amino acid substitutions identified in the spike region: N501T, Y453F, and S686G. N501T is positioned at the ACE2-interface in the spike receptor binding domain (RBD) and was identified in two different studies that used the same SARS-CoV-2 isolate for ferret inoculation [84,85]. The frequency of the N501T mutation in the viral population increased rapidly, becoming dominant in half of the ferret samples by 7 DPC [85]. The Y453F mutation is also located at the RBD-ACE2 interface and was found in all three infected ferrets in one study [87]. Y453F was also identified in SARS-CoV-2 isolates from mink in European mink farms [94,95,96]. The S686G mutation is positioned proximal to the novel SARS-CoV-2 furin cleavage site, and the frequency of this mutation increased rapidly in ferrets, becoming dominant at 1 DPC [85]. While these amino acid substitutions require further investigation, they support a potential trend of evolutionary pressure and adaptation of SARS-CoV-2 in the *Mustelidae* family that includes mink, ferrets, otters, and others [94,95,96].

Natural ferret infection has been demonstrated in Europe. A pet ferret tested positive for SARS-CoV-2, contracted from COVID-19 positive individuals, and exhibited gastrointestinal clinical signs [43]. In addition, a recent surveillance study in Spain found that 6 of 71 (8.4%) ferrets, kept as pets or working animals for rabbit hunting tested positive for SARS-CoV-2 viral RNA in nasal or rectal swabs and infectious virus was recovered from one rectal swab [91]. In addition, a seroprevalence study found that 2 out of 127 household ferrets (1.5%) kept as pets that were tested had antibodies against SARS-CoV-2 [92]. These results indicate that ferrets are indeed susceptible to natural SARS-CoV-2 infection. However, the number of households with ferrets is low compared to households with cats and dogs, and ferrets are rarely kept in large groups, in contrast to mink which are held on commercial fur farms. Interestingly, a recent study outlined a case in which two symptomatic COVID-19-positive individuals were in close and prolonged contact with 29 free-roaming pet ferrets, providing ample opportunity for natural transmission [173]. However, clinical samples failed to provide evidence of active ferret infection or seroconversion in this instance. The authors hypothesize that the N501T and S686G mutations are necessary for infection of ferrets, and that they were protected from the respective circulating human strain based on a genetic barrier [173]. Whether these spike mutations were present in naturally infected ferrets is of great interest and will require additional investigation.

Overall, ferrets are highly susceptible to experimental SARS-CoV-2 infection, which causes an acute disease with mild clinical signs; with the latter feature only observed in some instances. The disease progression is characterized by rapid onset of viral shedding that continues for several days, with infection localized to the upper respiratory tract. SARS-CoV-2 is readily transmitted between ferrets, with virus transmission via direct contact being more efficient than indirect aerosol transmission. Like another mustelid, the mink, potentially adaptive spike mutations are selected upon SARS-CoV-2 passage in ferrets. Moreover, ferrets are potentially susceptible to natural infection from humans, but the lower numbers of pet ferrets and lack of housing in large numbers make them of less concern. Regardless, ferret owners infected with SARS-CoV-2 should exercise caution and limit contact with their pets. Ferrets will likely continue to be an important preclinical animal model species for investigating SARS-CoV-2 and are currently being used as informative models to provide both fundamental insights into the virus pathogenesis [174,175,176], and for the pre-clinical development of therapeutics [177,178,179] and vaccines [180].

#### 2.1.6. Mink

American mink (*Neovison vison*) are highly susceptible to SARS-CoV-2 infection by both experimental and natural infection. Mink are the only species, besides humans, that have incurred population-level outbreaks of SARS-CoV-2 infection. Mink farms across Europe and North America have identified human-to-mink reverse zoonosis events and mink-to-mink transmission of SARS-CoV-2 resulting in clinical disease and mortality within mink populations. Most mink recover from infection, but their close proximity coupled with efficient viral transmission makes mink, and mink farms, particularly susceptible to spillover events. Mink are also the first species shown to infect humans (mink-to-human) and other animals, such as cats and wild mink, with SARS-CoV-2. Mink have infected humans with mink-adapted isolates containing mutations in the spike protein which were of significant concern to public health officials warranting destruction of many mink farms in The Netherlands and Denmark [96,97,181]. Overall, mink represent a significant host species for SARS-CoV-2, potentially complicating the current landscape of the SARS-CoV-2 ecology, and will require active surveillance and biosecurity considerations for the duration of the pandemic.

A single study showed that experimental inoculation of 13-month-old mink with a 5 × 10^6^ pfu SARS-CoV-2 dose administered intranasally resulted in productive infection [93]. Mink shed viable virus in nasal washes from 2 to 8 DPC with detection of viral RNA up to 12 DPC. Clinical signs were observed in mink, with 10–20% loss in body weight over 18 days and nasal discharge reported in one mink. However, no respiratory distress, change in behavior, increase in body temperature, or other clinical signs were reported. Histopathological lesions were present in the nasal cavities, trachea, and lungs of infected mink on 4 DPC. Infectious virus was isolated from nasal turbinate, soft palate, tonsil, and lungs, with only viral RNA detected in the trachea, submaxillary lymph node, and small intestine on 4 DPC.

Experimentally infected mink transmitted the virus efficiently through indirect contact (aerosol) to naïve animals in adjacent cages [93]. The contact mink became infected, shed infectious virus through the nasal cavity 5 to 11 days post exposure and exhibited weight loss, although at a lower level (5%) than principal inoculated mink. Importantly, no fatalities were reported in any of the nine principal infected or three contact mink, and all animals developed neutralizing antibodies by 18 DPC. This study clearly demonstrates that mink are highly susceptible to experimental SARS-CoV-2 infection with evidence of acute disease, clinical signs, efficient transmission by aerosols, and seroconversion.

As of September 2021, natural infection of farmed mink with SARS-CoV-2 has been reported in the USA, France, Italy, Spain, Denmark, Netherlands, Sweden, Canada, Greece, Poland, Lithuania, and Latvia [43]. The most detailed information has been reported from mink farms in The Netherlands and Denmark, which suffered widespread outbreaks from April to November 2020 that originated from SARS-CoV-2-infected humans [94,95,96,97,98,99]. These outbreaks were first identified by observation of clinical signs in mink and a higher-than-normal mortality rate, confirmed to be caused by SARS-CoV-2 infection [94,95,97,98,99]. SARS-CoV-2 RNA was widely detected in nasal, oral, and rectal swabs from mink on affected farms [94,95,96,98,99]. The most common clinical signs observed were nasal discharge, respiratory distress, and reduced activity and feed intake [95,97,98,99]. Reportedly, mink exhibiting severe clinical signs were consistently found dead within a matter of days [99]. Subclinical infections were also observed during these outbreaks, as large proportions of mink displaying no clinical signs tested positive for SARS-CoV-2 [97,99]. The lungs of naturally SARS-CoV-2-infected mink showed gross and histopathological changes, including diffuse interstitial pneumonia [95,99]. Moreover, the trachea and nasal conchae showed mild-to-severe histopathological changes in affected mink [99]. Viral RNA was consistently detected in the nasal conchae and lungs of infected mink, as well as in the liver and intestine of some mink [95]. The natural infection of farmed mink therefore mirrors the results from experimental infection, although the clinical signs were more severe and even mortality was observed in the affected farms, which is likely attributed to the large number of affected mink and/or the presence of comorbidities.

The spread of SARS-CoV-2 throughout mink farms was quite rapid, with an estimated outbreak duration of one month [99]. During this time, the infected mink developed a neutralizing antibody response with a seroprevalence greater than 95% in several farms tested [94,95,97,98]. On the respective farms, mink were generally housed individually in wire netting cages with solid sides in long rows [95]. Therefore, direct contact between mink is limited and transmission requires fomites or infectious droplets and/or aerosols. This is supported by evidence from mink farms in which SARS-CoV-2 was detected in both dust and air samples exhaled from mink within three meters from cages [94,95,97]. These data indicate SARS-CoV-2 spreads rapidly in mink farms via droplet/aerosol transmission, resulting in a limited period of widespread infection followed by herd immunity via neutralizing antibodies.

Importantly, phylogenetic evidence indicated that zoonotic mink-to-human transmission of SARS-CoV-2 occurred at both Dutch and Danish farms [94,95,96,98,182,183]. It was determined that between June and November 2020, 214 human cases of COVID-19 were identified in Denmark with SARS-CoV-2 variants associated with farmed mink [98,181]. The outbreaks at mink farms, therefore, represent the first evidence of a zoonotic event involving SARS-CoV-2 infected animals since the onset of the pandemic.

A troubling feature of the SARS-CoV-2 outbreaks on mink farms is that the number of mutations found in mink SARS-CoV-2 isolates is higher than what is typically found in humans, suggesting a selective pressure for viral adaptation in mink [94,95,96,184]. Mostly notably, the spike protein (S) accrued Y453F, F486L, and N501T substitutions in the receptor binding domain (RBD) and a G261D mutation in the N-terminal domain (NTD) of the S protein in samples from The Netherlands [95,98]. Amino acid substitutions were also found in the Danish mink SARS-CoV-2 isolates, with a viral lineage entitled cluster 5 (or ∆FVI-spike) presenting the following changes in the S protein: (i) the Y453F substitution (also found in the Dutch farms); (ii) a ∆H69V70 deletion in the NTD; (iii) a I692V substitution downstream of the novel furin cleavage site; (iv) a S1147L substitution close to the transmembrane domain; and (v) a M1229I substitution in the transmembrane domain [94,96]. The Y453F substitution is particularly interesting because amino acid Y453 in the S protein is highly conserved in SARS-related coronaviruses, and directly contacts a residue in the ACE2 receptor that differs between humans (H34) and mink and other mustelids (Y34); therefore, the Y453F substitution may be an adaptation to the mustelid ACE2 [182,185,186,187,188]. Moreover, a recent study indicated that the mink RBD mutations collectively increase the mean binding energy between the mutated mink spike protein and the mink ACE2 receptor, suggesting a potential adaptation of the virus to the new host [189]. The same study also suggests that the S protein mutations could modify the binding capacity of the mink SARS-CoV-2 isolates to the human ACE2 receptor and/or the efficacy of neutralizing antibodies, although further investigations are warranted [189]. The N501T substitution found in mink in The Netherlands also affects a residue which is directly in contact with the ACE2 receptor [187]. Likewise, an N501Y substitution has been identified in the Alpha, Beta, and Gamma VOC that are associated with higher transmission rates in humans [190,191,192]. While the mink (T501) and VOC (Y501) substitutions are different, their emergence suggests a selective pressure at the 501 residue of the S protein. Recent reports indicated that convalescent plasma from patients geographically isolated from the mink farms was less efficient at neutralizing the cluster 5/∆FVI-spike mink virus [182,188]. However, these additional investigations are required to fully characterize these mink-associated mutations as well as recently emerging VOC in humans. Regardless, there appears to be a distinct ability for SARS-CoV-2 to adapt to mink, as also observed in experimentally infected ferrets.

Lastly, SARS-CoV-2 was shown to have spread to cats, dogs, and wild mink in proximity to the farmed mink [95,97,193]. Both cats and dogs associated with mink farms tested positive for SARS-CoV-2 and had evidence of seroconversion [95,193]. Moreover, samples of flies collected at one mink farm, a swab from the foot of a seagull present within a mink farm, and fur from a harvested mink all tested positive for SARS-CoV-2 RNA [97]. In addition, the first documented SARS-CoV-2 infection in a wild animal occurred in December 2020 in Utah in a wild mink located near an infected mink farm [194]. Moreover, SARS-CoV-2 was detected in escaped mink from a farm associated with a SARS-CoV-2 outbreak in Utah, as well as in wild mink trapped in Spain 20 km from the nearest mink farms, indicating that wild and escaped mink could present a potential complication for biosecurity measures designed to contain the SARS-CoV-2 outbreaks on mink farms [195,196]. These results are of significant concern and vigilant surveillance and biosecurity measures will be required to isolate affected mink farms to avoid spillover into wild animal populations.

Mink have emerged as arguably the most significant domestic animal species involved in the current SARS-CoV-2 pandemic. Mink are highly susceptible to both natural and experimental SARS-CoV-2 infection and they exhibit clinical signs, including weight loss, pathological changes in the respiratory tract, and mortality. Evidence also suggests that SARS-CoV-2 readily adapts to mink and generates adaptive mutations at a higher rate than observed in humans. Lastly, mink readily transmit the virus during acute infection and have been shown to transmit to both humans and susceptible animal species such as cats, dogs, and wild mink. The role of farmed mink as a reservoir species capable of transmitting mutated SARS-CoV-2 strains to humans merits close surveillance and increased biosecurity measures. This is evidenced by the Danish government’s decision to cull all the country’s mink, including breeding stock, which amounted to 13 to 15 million animals [96,97,181]. Further work will be necessary to understand SARS-CoV-2 adaptation in mink and to understand the evolutionary pressures placed on the virus after passage in this host.

#### 2.1.7. Raccoon Dogs

Raccoon dogs (*Nyctereutes procyonoides*) have been shown to be susceptible to experimental infection with SARS-CoV-2 [100]. They develop a subclinical acute infection, primarily localized to the upper respiratory tract, with few histopathological lesions and a short period of viral shedding. Moreover, they are capable of viral transmission to naïve raccoon dogs, which is highly relevant considering they are farmed in large numbers for their fur.

Most raccoon dogs inoculated intranasally with a 10^5^ TCID_50_ virus dose developed a productive SARS-CoV-2 infection, although some of the animals (three out of nine) were believed not to have been successfully infected [100]. Shedding of infectious virus occurred during a relatively short period between 2 to 4 DPC from nasal and oropharyngeal cavities. Viral RNA was shed for a longer period, up to 16 DPC in nasal swabs, and was also detected in rectal swabs.

None of the inoculated or sentinel raccoon dogs in the study showed any overt clinical signs apart from lethargy observed in a few animals at 4 DPC [100]. Histopathological lesions indicative of mild rhinitis was present in infected raccoon dogs but not in the negative controls. Low levels of viral RNA were detected sporadically in the soft palate, tonsil, and brain. Interestingly, no viable virus or viral RNA was present in the lungs, but high levels of viral RNA were detected in the nasal conchae, and only low levels of viral RNA in other organs.

The raccoon dogs were also able to successfully transmit the virus and infect naïve animals in adjacent wire cages, a set-up meant to recapitulate conditions on farms. Most of the infected raccoon dogs developed antibodies to SARS-CoV-2 detected by indirect ELISA on 8 DPC, but low neutralizing antibodies were only detected in two of the nine animals by 8 or 16 DPC. Unfortunately, the low number of successfully infected raccoon dogs (six out of nine) and the early necropsy time points (4 and 8 DPC), which resulted in only one successfully infected animal remaining past 12 DPC, complicates conclusions regarding neutralizing immune responses in this species. No mutations in the viral sequence were observed after infection of raccoon dogs.

This single experimental study indicates that raccoon dogs are susceptible to SARS-CoV-2. Their susceptibility appears to be higher than domestic dogs, which are grouped in the same *Canidae* family, although they appear to shed the virus for only a short period of time, and exhibit limited clinical signs. Raccoon dogs are farmed for their fur and are housed in large numbers, with more than 14 million captive raccoon dogs estimated, primarily in China [197]. The study clearly indicates that raccoon dogs readily transmit the virus to naïve animals in conditions that recapitulate conditions on fur farms. Therefore, increased biosecurity and surveillance should be implemented at raccoon dog farms, despite the lack of current evidence for natural infection in this species.

#### 2.1.8. Cattle

Domestic cattle (*Bos taurus*) have been shown by two studies to have low susceptibility to experimental SARS-CoV-2 infection [101,102]. In one study, 4- to 5-month-old cattle were inoculated with a 10^5^ TCID_50_ dose of SARS-CoV-2 intranasally [101], whereas the other study inoculated 6-week-old calves either intratracheally or intravenously with a ~3 × 10^7^ TCID_50_ dose [102]. Both studies failed to demonstrate productive SARS-CoV-2 infection in cattle. The only evidence suggesting viral replication in the older cattle was the detection of viral RNA in nasal swabs in two out of six inoculated cattle on 2 to 3 DPC [101]. Virus isolation was not performed in this study and the viral RNA may have been residual material from challenge [101]. One of these older cattle developed low SARS-CoV-2 antibody titers, detected by indirect ELISA, but significant neutralizing antibodies were not observed [101]. Similar results were obtained for 6-week-old calves, with low-level detection of SARS-CoV-2 RNA in nasal swabs only on 3 DPC and 10 DPC from one intratracheally and intravenously inoculated calf, respectively [102]. Interestingly, a tracheobronchial lymph node from the intratracheally inoculated calf was positive at 9 DPC, but viable virus was not detected from any of these samples [102]. Some low neutralizing antibody titers were detected in calves at 7 DPC but were not detectable by 21 DPC [102]. All of the older cattle remained subclinical [101]; in contrast, most of the 6-week-old calves (five out of six) did exhibit periods of increased temperature and occasional coughing observed at 4 to 5 DPC; whether this was the result of SARS-CoV-2 infection cannot be ascertained [102]. No detailed pathology was performed on the older cattle [101], and no gross or histopathological lesions were observed in the 6-week-old calves apart from some minimal gross abnormalities in the kidney and liver {237]. Importantly, inoculated older cattle were unable to transmit SARS-CoV-2 to naïve cattle in direct contact [101].

These studies suggest that cattle have low susceptibility to SARS-CoV-2, with low levels of viral replication and limited seroconversion. Due to the large number of farmed cattle worldwide (over 1.5 billion), and their close association with humans, additional studies with different breeds and/or ages of cattle, and different virus isolates including VOC are warranted to further establish their level of susceptibility. Moreover, there is a lack of published information regarding other large, farmed ungulate species, including sheep, goats, camelids, or equids. Establishing the susceptibility and potential for transmission in these species is essential to limit infection of these animals or transmission of the virus through livestock herds, as well as to humans and susceptible wildlife species in close contact.

#### 2.1.9. Pigs

Domestic pigs (*Sus scrofa*) were found by several studies to be resistant or only marginally susceptible to SARS-CoV-2 infection [37,84,103,105,106]. The detection of viable SARS-CoV-2 virus or viral RNA in clinical samples from SARS-CoV-2-inoculated pigs was, at most, limited and sporadic. Moreover, clinical signs were rarely observed and none of the studies showed any transmission of virus to naïve contact pigs. Some of the studies did detect occasional low antibody responses; however, a strong neutralizing antibody response was only observed in pigs that were inoculated intravenously or intramuscularly.

Most studies used piglets ranging from 4 to 9 months old that were inoculated with a SARS-CoV-2 dose of 10^5^ to 10^6^ pfu either via: (i) nasal, tracheal, muscular, or venous administration [105]; (ii) combined oral/nasal/tracheal administration [103]; (iii) combined oral/nasal [104] administration; or (iv) nasal administration [37,84]. A recent study inoculated young (3-week-old) piglets with higher doses of 10^7^ pfu via intravenous administration or 2.5 × 10^7^ pfu via intranasal or intratracheal administration {238]. Detection of infectious SARS-CoV-2 or viral RNA in clinical samples was minimal in all studies. Two studies failed to detect any viral RNA in oropharyngeal, rectal, or nasal swabs [37,84]. Two studies only detected viral RNA in either nasal swab or trachea samples on 1 DPC, which may represent residual material from the inoculum [103,105]. One study detected viral RNA in nasal washes from two pigs and a mixed oral fluid sample on 3 DPC [104]. The recent study using a higher inoculation dose in young piglets did detect viral RNA in several clinical samples from multiple pigs, with most being positive between 1 to 3 DPC, which may also represent residual inoculum material [106]. Together, these results indicate that pigs generally do not develop a productive SARS-CoV-2 infection or readily shed virus.

In most of the studies, no pigs showed any clinical signs of infection, including increased temperature or respiratory distress [37,84,103,105,106]. However, in one study (10^6^ pfu administered via oral/nasal route) all pigs developed mild ocular and nasal discharge until 3 DPC, and one pig showed mild depression on 1 DPC with a cough maintained through 4 DPC [104]. No gross or microscopic pathological changes were observed in any of the principal inoculated pigs from any study. Interestingly, one study isolated viable virus from a submandibular lymph node on 13 DPC, which is the only sample from any pig study so far shown to contain viable virus [104]. Notably, the submandibular lymph node containing viable virus was collected from a pig exhibiting mild depression and a cough. Another study detected low levels of viral RNA in tonsil and lymph nodes from some pigs at 21 DPC [106]. Five of the studies included naïve pigs in direct contact with inoculated pigs, but no clinical signs were observed, and no viral RNA was detected for any of the contact pigs indicting transmission did not occur [37,84,103,104,106].

The antibody responses to SARS-CoV-2 in inoculated pigs differed between studies and varied based on the dose and inoculation route used. Two studies, which used a 10^5^ pfu dose intranasally, failed to detect any SARS-CoV-2 antibodies from inoculated pigs [37] or neutralizing antibodies [84]. Low levels of SARS-CoV-2 nucleocapsid-specific antibodies were detected in one study that used a 10^6^ TCID_50_ dose via nasal/tracheal administration, but reactivity decreased over time and may have been due to cross reactive maternal antibodies [103]. A different study that used a 10^6^ pfu dose via oral/nasal administration found weak neutralizing antibodies present in two principal inoculated pigs and in one oral fluid sample [104]. Another study that administered a 1 × 10^7^ pfu dose intravenously observed low levels of neutralizing antibodies on 7 DPC that diminished by 21 DPC, and only low neutralizing antibodies in some piglets receiving a 2.5 × 10^7^ pfu dose intranasally or intratracheally at 21 DPC [106] Lastly, one study that used a 6 × 10^5^ TCID_50_ dose administered intravenously or intramuscularly reported the detection of SARS-CoV-2 spike-specific antibodies by 14 DPC and neutralizing antibodies by 22 DPC [105]. Together, these results indicate that pigs can potentially mount a neutralizing antibody response against SARS-CoV-2, particularly after intravenous or intramuscular inoculation.

These studies indicate that pigs are only marginally susceptible to SARS-CoV-2 infection and are unable to transmit the virus to naïve animals in close contact. There was some evidence of viral replication in one study, most notably the detection of viable virus in one lymph node two weeks after infection [104], however, most of the studies failed to detect any productive SARS-CoV-2 infection. Importantly, all studies used pigs younger than 10 months old, therefore increased age may alter the susceptibility of pigs. Pigs are generally considered a strong pre-clinical candidate for infectious disease research based on similarities between the human and porcine anatomy and immune system [198]. However, pigs are obviously not a good candidate as a pre-clinical model for SARS-CoV-2, although intravenous/intramuscular injection may be a useful method to analyze SARS-CoV-2 specific antibody responses to vaccine candidates. There are currently no reports of natural human-to-pig infections; therefore, the > 600 million farmed pigs globally represent a low possibility of developing into an important amplifying host for SARS-CoV-2, and the biosecurity and surveillance requirements for SARS-CoV-2 in this species are therefore negligible.

#### 2.1.10. Poultry

Chickens (*Gallus gallus domesticus*) were found by multiple studies to be completely resistant to SARS-CoV-2 infection [37,84,107,108]. Moreover, turkeys (*Meleagris gallopavo domesticus*), Japanese quail (*Coturnix japonica*), Pekin ducks (*Anas platyrhinchos domesticus*), and white Chinese geese (*Anser cygnoides*) were also found to be resistant to SARS-CoV-2 infection [37,107]. Two- to six-week-old poultry species were infected with a 7 × 10^4^ to 1 × 10^6^ pfu dose of SARS-CoV-2 either intranasally [37], choanally [107], or orally/nasally/ocularly [84,108]. No viral RNA was detected in any clinical samples [37,84,107,108], and no clinical signs or gross/histopathological lesions were observed [84,107,108]; also, no viral RNA was detected in any tissues collected [108]. Moreover, no SARS-CoV-2-specific or neutralizing antibodies were detected for any species in any of the studies. Chickens and ducks were unable to transmit the virus to naïve animals [37,84]. Moreover, multiple studies determined that embryonic chicken eggs were also resistant to SARS-CoV-2 infection [84,107,108,199]. These results clearly indicate that chickens and other farmed poultry species are not susceptible to SARS-CoV-2. It is therefore not surprising that no documented cases of human-to-poultry transmission of SARS-CoV-2 have been reported. The SARS-CoV-2 surveillance and biosecurity efforts associated with poultry farming are of very low concern.

### 2.2. Wild/Peridomestic Animals

Several studies have outlined SARS-CoV-2 susceptibility of wild species that are peridomestic, i.e., occasionally live in proximity to or come in contact with humans. The susceptibility of these animals is crucial for understanding the disease ecology of SARS-CoV-2 and the potential for the establishment of wild amplifying hosts or reservoir species that could complicate mitigation efforts.

#### 2.2.1. Deer Mice

North American deer mice (*Peromyscus maniculatus*) have been shown by multiple studies to be susceptible to experimental SARS-CoV-2 infection [83,109,110]. Deer mice are widely distributed across North America and are members of the *Cricetidae* family, along with hamsters. Deer mice are also notable as reservoirs for several human diseases, including *Borrelia burgdorferi* (the etiological agent of Lyme disease) and Sin Nombre orthohantavirus. Deer mice generally exhibit a subclinical infection in the respiratory tract, although some weight loss was also noted. Deer mice are also capable of transmitting the virus over multiple passages and develop a robust neutralizing antibody response.

Experimental studies investigating the susceptibility of deer mice to SARS-CoV-2 have used a 4 × 10^4^ to 1 × 10^5^ TCID_50_ viral dose administered intranasally [83,109,110]. One of the studies used wild, trapped deer mice [83], whereas the others used in-house colonies, aged 2 weeks to 8 months [109,110].

All experimental inoculations of deer mice resulted in productive SARS-CoV-2 infection [83,109,110]. Viable virus was isolated from oral and rectal swabs between 1 and 4 DPC with some low titer isolation in rectal swabs up to 8 DPC [83,110]. Viral RNA was also consistently detected in oral and rectal swabs, with extended detection in oral swabs up to 21 DPC in one study [109]. Moreover, viral RNA was detected in a fecal sample collected on 4 DPC and one urine sample on 6 DPC [110].

Two of the studies did not report any clinical signs in SARS-CoV-2-infected deer mice, except for occasional ruffled fur in some animals [83,110]. However, weight loss for 4 to 6 days was observed in one study [109], before normal weight was regained, similar to what is observed for SARS-CoV-2-infected hamsters [71,72,73,74,75,76,77,79]. Mild-to-moderate histopathological lesions in the respiratory tract were observed in infected deer mice that resolved after the period of acute infection [83,109], and one study outlined histopathological lesions in the olfactory epithelium and brain [109]. Infectious virus was isolated from the nasal turbinate from 2 to 4 DPC, from most trachea samples on 3 DPC, and from the lung between 2 to 6 DPC [83,109,110]. Low levels of infectious virus were also isolated from colon and small intestine samples from 2 to 4 DPC [110]. Interestingly, viral RNA remained in the nasal turbinate, lung, and intestine up to 21 DPC [110]. Viral RNA was also detected in the blood of deer mice from 1 to 3 DPC [110].

A robust neutralizing antibody response against SARS-CoV-2 was found in infected deer mice as early as 14 DPC [83,109,110]. Infected deer mice were able to successfully transmit SARS-CoV-2 to naïve deer mice that were placed in the same cage in direct contact at 1 DPC; transmission resulted in a similar disease progression in the contact deer mice and the generation of neutralizing antibodies, although the observed weight loss was less in the contact deer mice [109,110]. Moreover, one study found that the contact deer mice (P1) were also able to transmit the virus to another group of naïve deer mice (P2) in direct contact, indicating that transmission between deer mice can be sustained through multiple passages [109]. Lastly, this transmission study showed a twelve-nucleotide insertion in the N-terminal domain of the spike protein in SARS-CoV-2 isolated from the P2 generation deer mice that was minimally present in the challenge material [109]. This results in a Lysine-Leucine-Arginine-Serine (KLRS) insertion in a predicted solvent exposed loop in the N-terminal domain of the S protein [109]. Whether this insertion is the result of a transmission bottleneck and/or confers a selective advantage requires further investigation.

Although no natural infection of wild deer mice has been reported, these studies indicate that this widely dispersed wild rodent can become infected with SARS-CoV-2 and efficiently spread the virus to naïve animals. Moreover, predation of deer mice by susceptible species, such as cats, may be a potential means of transmission. Deer mice could be used as a viable small animal model for SARS-CoV-2 research, however they lack the significant resources and standardization currently available for Syrian golden hamsters. Surveillance of wild species of *Cricetidae* rodents is warranted, particularly those in close contacts with human habitations or in close contact with infected mink farms.

#### 2.2.2. Bushy-Tailed Woodrats

Bushy tailed woodrats (also known as packrats, *Neotoma cinerea*) have been shown by a single study to be susceptible to SARS-CoV-2 [83]. They are also members of the *Cricetidae* family and are widely distributed over various habitats in the western US and Canada. A SARS-CoV-2 dose between 3 × 10^4^ and 8 × 10^4^ pfu per animal was administered nasally to wild, trapped woodrats. Inoculation resulted in a productive infection, with infectious virus shed orally from 1 to 5 DPC. None of the animals displayed any clinical signs throughout the course of infection, including altered temperature, weight loss, or change in behavior. However, mild histopathological lesions were observed in the lungs of some of the woodrats during the period of acute infection (3 DPC). Infectious virus was detected in a proportion of nasal turbinate, trachea, and lung samples on 3 DPC. Neutralizing antibodies were detected in the woodrats at 28 DPC. The ability for woodrats to transmit the virus to naïve animals was not tested.

Woodrats, like other members of the *Cricetidae* family, are clearly susceptible to SARS-CoV-2 and develop a similar disease progression, although clinical signs such as weight loss were not reported. No instance of natural infection of SARS-CoV-2 has been documented in wild woodrats. However, active surveillance should be performed for this species in a similar manner as for deer mice based on their peridomestic association with human dwellings, and potential association with susceptible farmed (mink, deer) or predatory (cats) species. Their potential use as a research model is limited, compared to hamsters or deer mice, based on the lack of active research colonies and clinical signs.

#### 2.2.3. House Mice, Fox Squirrels, Wyoming Ground Squirrels, and Black-Tailed Prairie Dogs

Wild-caught rodents consisting of house mice (*Mus musculus*), fox squirrels (*Sciurus niger*), Wyoming ground squirrels (*Urocitellus elegans*), and black-tailed prairie dogs (*Cynomys ludovicianus*) were shown by a single study to be resistant to experimental infection with SARS-CoV-2 [83]. Each species was administered between 3 × 10^4^ and 8 × 10^4^ pfu SARS-CoV-2 nasally. This failed to result in a productive infection in any of these species, with no detection of infectious virus in swabs or tissues and no observation of clinical signs or pathological changes. No neutralizing antibodies were detected in house mice or prairie dogs, which were the only species tested for antibody response. These data, therefore, suggest that these widely dispersed rodents in the *Muridae* (house mice) and *Sciuridae* (fox squirrels, Wyoming ground squirrels, black-tailed prairie dogs) families are not susceptible to ancestral SARS-CoV-2 infection, in contrast to their *Cricetidae* family relatives. The situation, however, may be different when VOC are used for infection. As mentioned below, non-transgenic mice are resistant to the ancestral SARS-CoV-2 strains but susceptible to SARS-CoV-2 variants containing the N501Y polymorphism in their S gene [140]. Therefore, active surveillance should be performed for *Muridae* species whereas the *Sciuridae* are of lower concern and do not require active surveillance.

#### 2.2.4. Otters

A group of Asian small-clawed otters (*Aonyx cinereus*) at a zoo in the US were confirmed to be infected with SARS-CoV-2 and are thus susceptible to natural infection that was presumed to be from an asymptomatic caretaker [111]. Otters are members of the *Mustelidae* family; therefore, it is not surprising that they are susceptible to disease. The otters exhibited clinical signs, including sneezing, nasal discharge, mild lethargy, and coughing. Reports indicate that the otters were geriatric, which may have exacerbated the clinical signs seen in these animals [112]. Asian small-clawed otters reportedly live in groups of up to 15 to 20 individuals [200], which provides conditions for spread via direct contact as has been reported in experimental and natural infections of other mustelid species. Infection of otters underscores the importance of monitoring and practicing caution around mustelid species in both captive and wild environments to avoid introduction of SARS-CoV-2 into susceptible populations.

#### 2.2.5. Striped Skunks and Raccoons

Striped skunks (*Mephitis mephitis*) and raccoons (*Procylon lotor*) are both categorized within the *Musteliodea* superfamily that also includes SARS-CoV-2-susceptible ferrets, otters, and mink. Interestingly, a single study showed that skunks are susceptible to SARS-CoV-2 infection, whereas raccoons are not [83].

Skunks and raccoons obtained from a private vendor were administered between 3 × 10^4^ and 8 × 10^4^ pfu SARS-CoV-2 per animal intranasally, which resulted in a productive SARS-CoV-2 infection in four out of six inoculated skunks. Infectious virus was shed by a proportion of skunks in oral swabs between 2 and 5 DPC and nasal swabs between 2 and 7 DPC. Despite clear viral shedding, none of the skunks exhibited any clinical signs for the duration of the study and no significant gross or histopathological lesions were observed. Infectious virus was isolated from the nasal turbinate of two of the skunks on 3 DPC, including one that did not exhibit viral shedding in swab samples. All productively infected skunks developed neutralizing antibodies by 28 DPC. In contrast, no infectious virus was detected in any inoculated raccoon clinical samples or tissues for the duration of the study and there was no evidence of seroconversion.

These results indicate that skunks are susceptible to SARS-CoV-2 infection and that raccoons are not. Skunks were individually housed, therefore any insights into the potential for transmission was not determined, although the level of viral shedding would indicate this is possible. Both skunks and raccoons are widely dispersed, are commonly associated with human dwellings, and do have some direct interactions with humans and susceptible pet/wild species. Active surveillance of skunks should therefore be included in any SARS-CoV-2 wildlife monitoring program, whereas raccoons should be considered of very low concern.

#### 2.2.6. White-Tailed Deer

White-tailed deer (*Odocoileus virginianus*) have been shown in two different studies to be susceptible to experimental infection with SARS-CoV-2 [113,114]. Experimental infection occurred in both fawns and adult white-tailed deer and resulted in subclinical infections with a period of viral shedding from nasal and oral secretions and in fecal samples. Infected white-tailed deer were able to successfully transmit SARS-CoV-2 to naïve animals via aerosols and direct contact, and all deer generated strong neutralizing antibodies against the virus. Moreover, recent evidence indicates that wild white-tailed deer are susceptible to natural infection [115,116], which will require additional surveillance to determine if they have become a reservoir for SARS-CoV-2.

Each experimental study investigated SARS-CoV-2 infection in white-tailed deer in distinct age cohorts. One study inoculated 6-week-old fawns with approximately 10^7^ TCID_50_ SARS-CoV-2 intranasally [114], whereas the other study inoculated 2-year-old adult deer with 10^6^ TCID_50_ orally and nasally simultaneously [113].

Both studies resulted in productive SARS-CoV-2 infection in white-tailed deer. Infectious virus was detected in nasal secretions from inoculated deer between 1 and 5 DPC, one oral swab at 3 DPC, rectal swabs at 5 DPC, and fecal samples at 1 DPC [113,114]. Infectious virus was also detected in one adult deer from bronchoalveolar lavage fluid and nasal wash on 4 DPC [113]. Viral RNA was generally detected for a longer period; from 1 to 7 DPC in adult nasal swabs and 1 to 21 DPC in fawns [113,114]. Detection of viral RNA in oral and rectal swabs was comparatively lower and more sporadic in both studies. These results indicate that both adult and juvenile white-tailed deer are clearly susceptible to productive SARS-CoV-2 infection.

Both age cohorts of white-tailed deer remained generally subclinical throughout the studies, except for a slight elevation in body temperature recorded in both adult and juvenile deer between 1 and 3 DPC [113,114], and isolated ocular or nasal discharge between 5 to 10 DPC in adult deer [113]. Two inoculated adult deer were humanely euthanized at 4 DPC and histopathological evaluation revealed rhinitis, marked attenuation of the respiratory epithelium of the trachea, bronchitis, and in some cases bronchiolitis, but no interstitial pneumonia [113]. One of the inoculated fawns died due to an unrelated intestinal perforation on 8 DPC but exhibited no gross or histopathological lesions indicative of a SARS-CoV-2 infection [114]. All remaining deer euthanized at 18 or 21 DPC lacked any significant lesions related to a respiratory infection [113,114].

Viable SARS-CoV-2 virus was isolated only from the trachea and bronchi of one of the two adult deer euthanized on 4 DPC during the period of acute infection [113]. However, SARS-CoV-2 RNA was widely detected in respiratory tissues at 4 DPC, as well as in tissues such as tonsil, various lymph nodes, spleen, liver, heart, kidney, bone marrow, stomach, brain, and olfactory bulb [113]. On 8 DPC, SARS-CoV-2 RNA was detected in the nasal turbinate, tonsil, spleen, digestive tract, and various lymph nodes of the juvenile animal [114]. By 18 or 21 DPC, detection of viral RNA was significantly diminished, remaining mostly in the upper respiratory tract, tonsil, and isolated lymph nodes [113,114].

White-tailed deer were also able to efficiently transmit SARS-CoV-2 to naïve animals via both direct [113] and indirect [114] contact. Infectious virus was detected in nasal swabs from sentinel fawns in indirect contacts between 2 and 7 DPC with viral RNA persisting up to 14 DPC [114]. Viral RNA was also detected in nasal swabs in adult sentinel deer in direct contact between 3 to 10 DPC [113]. More sporadic detection of SARS-CoV-2 RNA was detected in rectal or oral swabs [113,114]. SARS-CoV-2 RNA was detected in sentinel deer at 18 or 21 DPC in the nasopharynx, nasal turbinates, tonsil, lymph nodes, and bone marrow [113,114]. Despite the evidence of active infection, contact deer remained subclinical throughout the study.

Consistent with these results, both principal inoculated and contact deer developed a robust neutralizing antibody response by 7 DPC [113,114]. In both studies, the level of neutralizing antibodies increased over the course of the study, with high levels of neutralizing antibodies detected at 18 or 21 DPC.

Of the six adult deer that were infected, five were found to be pregnant, which provided a unique opportunity to examine vertical transmission of SARS-CoV-2 between the pregnant animal and fetuses [113]. One of the two principal infected deer euthanized on 4 DPC was pregnant, and two of the three fetuses from this deer had detectable levels of SARS-CoV-2 RNA. Interestingly, none of the fetuses (*n* = 9) collected on 18 DPC had detectable levels of SARS-CoV-2 RNA. Over 50% of the fetuses collected at 18 DPC were non-viable, although the specific role of SARS-CoV-2 in this outcome is difficult to ascertain under the experimental conditions used. Regardless, these data indicate that vertical transmission of SARS-CoV-2 is possible during the period of acute infection and additional studies are necessary to determine the overall effect of vertical transmission during SARS-CoV-2 infection.

One of the studies [113] performed a competitive viral replication experiment in which adult deer were infected with approximately equal amounts of ancestral lineage A virus (USA-WA1/2020) and the recently emerged Alpha VOC (B.1.1.7 lineage) isolate [201]. It was determined that the Alpha variant had a replicative advantage over the ancestral lineage A isolate in white-tailed deer, as demonstrated by the virus shed from nasal and oral cavities and present in tissues, which is consistent with the selective advantage of the Alpha VOC in human populations [202,203].

The first evidence of natural infection of white-tailed deer was a recent sero-surveillance study in several U.S. states that indicates there may be significant levels of exposure in wild deer populations [115]. Evaluation of 624 pre- and post-pandemic serum samples from white-tailed deer revealed SARS-CoV-2-specific antibodies in 40% of the samples from 2021, suggesting that white-tailed deer have been exposed to SARS-CoV-2 in the wild. While the cross-reactivity from other coronaviruses cannot be definitively ruled out, the high level of neutralizing antibodies found in many of the samples are indicative of exposure to SARS-CoV-2. In addition, recently white-tailed deer have been confirmed to test RT-qPCR positive for SARS-CoV-2, providing further evidence of the susceptibility of this wild animal species [116]. These results are of significant concern and warrant additional surveillance studies to identify deer populations that have been exposed to SARS-CoV-2 and the extent of maintenance of the virus in these wild populations.

Cumulatively, these studies indicate that wild as well as farmed young and adult white-tailed deer are susceptible to productive SARS-CoV-2 infection and can efficiently transmit the virus to naïve animals. Although humans and deer rarely come into direct contact in the wild (except for hunters), farmed deer are common and humans at these farms should implement additional biosafety procedures and surveillance to avoid introduction into farmed deer herds. In addition, wildlife monitoring must include white-tailed deer to ensure that the virus is not becoming established in the large numbers of white-tailed deer in North America and to determine the extent of SARS-CoV-2 circulation in wild populations already.

#### 2.2.7. Tree Shrews

Tree shrews (*Tupaia belangeri*) have been shown in two different studies to be moderately susceptible to experimental SARS-CoV-2 infection [117,118]. Tree shrews are small mammals native to Southeast Asia that are attractive as clinical models based on their evolutionary relationship to primates [204]. Most experimentally inoculated tree shrews become infected with SARS-CoV-2 with clear pathological manifestations of respiratory disease and viral RNA present in a variety of tissue types. However, tree shrews lack obvious clinical signs and consistent viral shedding, limiting their usefulness as a model organism for SARS-CoV-2 research.

Tree shrews were experimentally infected with either a 10^6^ TCID_50_ dose per animal of SARS-CoV-2 intranasally [117] or a 10^7^ TCID_50_ dose per animal via oral, nasal, and ocular routes [118]. A wide age range of animals, from six months- to seven years old, were used in these studies [117,118].

Unfortunately, infectious virus isolation from clinical swabs was not performed in either study [117,118]. Moreover, one study did not detect any viral RNA in clinical swabs from any tree shrews between 3 and 12 DPC [117]. However, one study clearly detected SARS-CoV-2 RNA in nasal, oral, and rectal swabs in a proportion of tree shrews at a relatively late time point post infection, between 6 and 12 DPC [117]. However, not all the tree shrews shed viral RNA and RNA detection was highly sporadic [117]. Younger tree shrews shed viral RNA earlier, mainly between 6 to 8 DPC, whereas old tree shrews shed mostly between 8 to 12 DPC [117].

The only clinical signs observed in experimentally inoculated tree shrews was an increase in body temperature in a proportion of animals that peaked at 6 to 8 DPC [117]. Importantly, mild-to-moderate histopathological lesions were observed in the respiratory tract in both studies [117,118]. Pulmonary infiltrates were observed in X-rays between 3 and 14 DPC [118], suggesting a viral respiratory disease. Consistent with these data, infectious virus was isolated from the trachea and lung of three different tree shews on 4 and 7 DPC [117]. Moreover, viral RNA was detected in the lungs of most inoculated tree shrews between 3 and 14 DPC [117,118]. Thus, despite only sporadic viral shedding in clinical samples, tree shrews were clearly infected with SARS-CoV-2. Additionally, histopathological changes were documented in several non-respiratory tissues in one study, including the intestine, pancreas, heart, spleen, conjunctiva, and brain [117]. Accordingly, SARS-CoV-2 RNA was also detected in a range of non-respiratory tissues [117,118]. This unique pattern of histopathological changes and viral RNA detection in non-respiratory tissue suggests a possibly novel tropism of SARS-CoV-2 in tree shrews. Interestingly, infectious virus was isolated from the pancreas of one tree shrew on 4 DPC, which is the only documented involvement of this organ in SARS-CoV-2 infections in animals, thought pancreatic infection of SARS-CoV-2 has been demonstrated in humans [205].

One of the two studies reported that neutralizing antibodies were detected in infected tree shrews, but the antibody titer and the DPC were not reported [118]; neutralizing antibody response was not tested in the other study. Moreover, no transmission study was performed; therefore, the ability of tree shrews to maintain the virus in animal populations remains unknown.

Overall, tree shrews appear to be moderately susceptible to SARS-CoV-2 infection and generally demonstrate a subclinical and self-limiting disease. However, the absence of consistent viral shedding in tree shrews and the lack of data regarding transmissibility complicates conclusions regarding their ability to become productively infected with SARS-CoV-2. The mild disease manifestations and variable patterns of viral detection in clinical samples of tree shrews limit their usefulness as SARS-CoV-2 animal models. However, the unique involvement of the pancreas in this species may provide some insights into diabetes-related complications of COVID-19 in humans. The moderate susceptibility observed in tree shrews and the lack of consistent viral shedding makes this species of limited concern for wildlife surveillance programs.

#### 2.2.8. Megachiroptera Bats

Egyptian fruit bats (*Rousettus aegyptiacus*), a Megachiroptera species, have been shown to be susceptible to experimental SARS-CoV-2 infection [84]. After intranasal infection, fruit bats exhibit a sub-clinical infection localized to the upper respiratory tract. Transmission between bats was observed, but was inefficient, and SARS-CoV-2 inoculation only elicited a weak neutralizing antibody response.

Fruit bats, approximately 1 to 5 years of age, were infected with a 10^5^ TCID_50_ dose per animal of SARS-CoV-2 intranasally [84]. Inoculation of the fruit bats resulted in a productive SARS-CoV-2 infection in the inoculated bats. Viral RNA was detected in oral swabs in all inoculated bats between 2 and 12 DPC, but infectious virus was isolated from only one oral swab on 2 DPC. Moreover, SARS-CoV-2 RNA was detected in fecal samples from all three bat cages on 2 and 4 DPC.

Despite an active SARS-CoV-2 infection, no clinical signs were observed in any of the bats, including fever or weight loss, for the 21-day duration of the study [84]. Only mild-to-moderate histopathological changes were observed in the nasal epithelium between 4 and 8 DPC, and mild histopathological changes were observed in the lungs of some animals between 4 and 12 DPC. Infectious virus could only be isolated from the nasal turbinates and trachea of one bat on 4 DPC. Viral RNA was most consistently detected in the nasal turbinates between 4 and 21 DPC, and in skin, trachea, lymph node, lung, heart, adrenal gland, and duodenum samples at 4 and 8 DPC.

Infected fruit bats successfully transmitted SARS-CoV-2 to one of three naïve bats that were co-housed in direct contact starting on 1 DPC [84]. Therefore, transmission between bats is possible, though somewhat inefficient. Moreover, the contact fruit bat that became infected was pregnant; therefore, transmission may have been influenced by immunosuppression associated with pregnancy. All principal inoculated bats, and the one infected contact bat, developed a weak neutralizing antibody response against SARS-CoV-2 starting at 8 DPC; apparently low antibody titers in bats are typical [206].

These results indicate that *Megachiroptera* fruit bats are susceptible to SARS-CoV-2 infection. While they are not an ideal clinical model, wild populations are at risk of becoming infected by humans or infected other animals in their environment, and caution should be exercised to avoid potential disease-transmitting interactions between humans and bats during the pandemic.

#### 2.2.9. Microchiroptera Bats

North American big brown bats (*Eptesicus fuscus*), a *Microcheroptera* species, were found to be resistant to experimental SARS-CoV-2 infection in a single study [119]. This species is common in North America, and they often roost and hibernate in man-made structures in proximity to humans. The bats in this study (ages unknown) were obtained during hibernation from human-made dwellings and were inoculated orally/nasally with 10^5^ TCID_50_ SARS-CoV-2 per animal and co-housed in pairs with an uninoculated bat. Prior to inoculation, five of the sixteen bats tested positive for an Alphacoronavirus infection. SARS-CoV-2 inoculation in big brown bats did not result in a productive infection. SARS-CoV-2 RNA was not detected in oral or rectal swabs in any of the bats for the 20-day duration of the study. Moreover, no clinical signs were observed, and no pathological signs indicative of SARS-CoV-2 infection were reported. Additionally, no viral RNA was detected in any tissues collected during necropsies on 6, 12, or 20 DPC and no SARS-CoV-2-reactive antibodies were detected for any of the bats. There was also no transmission to uninoculated bats throughout the study. These results indicate that this common North American *Microchiroptera* bat species is wholly resistant to infection with SARS-CoV-2 under the experimental conditions. Due to their importance as reservoir species and their association with persistent coronavirus infection and transmission, additional susceptibility studies with *Microchiroptera* bat species should be pursued, possibly using structure-guided studies of bat ACE2 sequences to identify other *Microchiroptera* species that may be susceptible to infection. Based on this study, big brown bat species should be currently regarded as low concern for surveillance and monitoring for SARS-CoV-2 infection.

#### 2.2.10. Non-Human Primates

Numerous studies have demonstrated that several non-human primate (NHP) species are susceptible to SARS-CoV-2 infection. The Old-World monkey family members rhesus macaques (*Macaca mulatta*), cynomolgus macaques (*Macaca fascicularis*), African green monkeys (*Chlorocebus aethiops*), and baboons (*Papio hamadryas*), and the New World monkey family member common marmosets (*Callithrix jacchus*), have all been shown to be susceptible to experimental SARS-CoV-2 infection to varying degrees. In general, each of these species exhibits minimal-to-moderate self-limiting clinical manifestations of SARS-CoV-2 infection. NHPs therefore remain important model species for studying SARS-CoV-2 virulence and host immune responses, and for the development of vaccines and therapeutics. While natural human-to-NHP infection has not been documented in these species, a group of Western lowland Gorillas (*Gorilla gorilla*) at a zoo were inadvertently infected by a caretaker [137], demonstrating that natural infection is possible for NHP species.

Rhesus macaques have been the most extensively studied NHP species regarding experimental SARS-CoV-2 infection [120,121,122,123,124,125,126,127,128,129,130,131]. A wide range of age cohorts (<1 to 22 years old), dose ranges (~1 × 10^4^ to 5 × 10^6^ pfu) and administration routes (combination nasal/tracheal/ocular/oral/venous) have been used, each resulting in productive SARS-CoV-2 infection. After inoculation, infectious virus was detected in nasal swabs between 1 and 5 DPC [125,128,131] and oral swabs between 1 and 6 DPC [125], defining the period of acute viral shedding. One study also isolated infectious virus from a single rectal sample on 9 DPC [120]. Experimental SARS-CoV-2 infection in rhesus macaques can be completely subclinical [131], but is more often accompanied by mild, transient clinical signs, including elevated body temperatures [120,122,128,129,130], decreased activity [120,124,126,128], appetite [120,123,124,125,129] or body weight [122,123,125,126,129,130], or changes in respiratory patterns [123,129,130]. Pathological changes also occurred in the respiratory tracts of SARS-CoV-2-infected rhesus macaques, with mild-to-moderate interstitial pneumonia consistently reported between 2 and 12 DPC that generally resolves after this period [120,122,123,124,125,126,127,128,129,131,132]. Infectious virus was isolated from lung samples and bronchus from infected rhesus macaques on 3 DPC [125,128,129]. Viral RNA was detected in a wider variety of tissue samples apart from the respiratory tract, including nervous, lymphatic, gastrointestinal, and urogenital tissues, liver, and heart [120,122,123,124,125,126,127,128,129,130,131], although these were not consistent between studies or between animals within individual studies. Neutralizing antibodies were detected in infected rhesus macaques as early as 8 DPC [120,122,123,124,125,127,129,131], and several studies have shown protection from re-infection [123,124,125].

Cynomolgus macaques also become productively infected upon experimental SARS-CoV-2 inoculation, providing an alternative NHP model to rhesus macaques. Relatively high doses have been used to infect cynomolgus macaques, ranging from approximately 1 × 10^6^ to 2 × 10^7^ pfu SARS-CoV-2, administered via various combinations of tracheal, oral, ocular, nasal, and venous routes [122,128,131,133]. Infectious virus was isolated from nasal and oral swabs between 1 to 7 DPC and conjunctival swabs between 1 and 3 DPC [128,133]. Clinical signs observed in cynomolgus macaques was generally milder than in rhesus macaques, although transient increases in body temperature and decreases in appetite and body weight were observed, [122,128,131,133]. Mild, self-limiting histopathological changes in the respiratory tract, including interstitial pneumonia, were observed [122,128,131,133]. Infectious virus was isolated from lung samples on 3 DPC [128], with viral RNA being detected in the entire respiratory tract at 4 to 5 DPC [131] and in the bronchus, stomach, and spleen at 13 DPC [122]. Most cynomolgus macaques developed neutralizing antibodies as early as 7 DPC [122,128,131].

African green monkeys (AGMs) have also been shown by several studies to be susceptible to productive experimental SARS-CoV-2 infection and to be a good model for pre-clinical SARS-CoV-2 studies [132,134,135,136]. A wide age cohort (3.5 to 16 years old) of AGMs were used in these studies, a wide range of doses (1.5 × 10^3^ to 2.5 × 10^6^ pfu), as well as different inoculation routes: aerosol [132,134], intranasal atomized particle delivery [135], tracheal/nasal [136], oral/nasal/tracheal/ocular [132,134]). Infectious virus was isolated from nasal swabs between 2 and 7 DPC [134,135,136], oral swabs between 2 to 9 DPC [134,135,136], and some rectal swabs between 2 and 5 DPC [134,136]. Interestingly, one study also found a resurgence of virus shedding in some rectal and nasal/oral swab samples at 14 and 21 DPC, respectively [134]. Most AGMs demonstrated mild, transient clinical signs, including decreased appetite [135,136], anorexia [135], elevated body temperatures [134], and changes in respiratory rate [134]. Interestingly, in one study, two AGMs developed tachypnea that progressed to severe respiratory distress, hypothermia, and low oxygen saturation on 8 and 22 DPC, respectively, prompting humane euthanasia that revealed severe consolidation and edema in the lungs, consistent with interstitial pneumonia [132]. Apart from these notable instances of ARDS, all other AGMs had generally mild interstitial pneumonia during the acute infection period around 5 DPC that began to resolve thereafter [132,134,135,136]. Consistent with this, infectious virus was detected in the lung of all AGMs in one study at 5 DPC [136]. Neutralizing antibodies were detected as early as 5 DPC [134,135,136], and AGMs were found to be resistant to re-infection [136].

Baboons were also found in a single study to be susceptible to productive infection with SARS-CoV-2 [127]. Young (two-year-old) and old (10- to 20-year-old) baboons were inoculated with approximately 10^6^ pfu SARS-CoV-2 via a combination of tracheal/nasal/ocular administration. Although virus isolation was not performed during the acute infection period, SARS-CoV-2 RNA was detected in the nasal and rectal swabs of a proportion of inoculated baboons from 3 to 17 DPC, with a higher amount detected in older baboons. Although no clinical signs were reported, chest X-rays revealed a higher level of lung inflammation in baboons compared to rhesus macaques, particularly at 3 to 6 DPC. Moreover, gross and histopathological lung lesions were observed at 14 and 17 DPC, indicating that baboons develop a more severe and long-lasting disease than rhesus macaques. Viral RNA was detected in the lungs of baboons at 14 and 17 DPC, but no subgenomic RNA was detected, indicating an absence of replicating virus at this time point. Further studies will be necessary to more fully determine SARS-CoV-2 pathogenesis in baboons, such as the neutralizing antibody response and potential for transmission, but this species could clearly make a useful NHP research model.

Common marmosets have been shown by two studies to be susceptible to SARS-CoV-2 infection [122,127], although with a much milder disease course compared to the Old-World monkey species. Approximately 10^6^ pfu SARS-CoV-2 was administered to marmosets via tracheal/nasal/ocular route in both studies. One study inoculated only older (6 to 11 years old) marmosets [127], and the age was not specified in the other study [122]. One of the studies showed consistent detection of SARS-CoV-2 RNA in nasal swabs from 2 to 12 DPC, in oral and rectal swabs from 2 to 10 DPC, in blood from 2 to 8 DPC, and in feces of one marmoset from 6 to 21 DPC [122]. Interestingly, these results contrast with the other study, in which viral RNA was only detected in the nasal cavity of some marmosets at 3 and 6 DPC and was not detected in oral swabs at any point. Marmosets remained mostly subclinical in both studies, with an increase in body temperature only noted in 3 out of 6 marmosets in one study [122]. Marmosets also showed only mild respiratory pathology, with some histopathological lesions in the lung at 3 and 14 DPC [122,127] and some histopathological lesions in liver and spleen noted [122]. Viral RNA was detected in the lung of one out of two marmosets at 3 DPC and two out of four marmosets at 14 DPC in one study [127], whereas no viral RNA was detected in any tissues tested on 13 DPC in the other study [122]. Only one of the studies tested for the presence of neutralizing antibodies and failed to detect them by 21 DPC [122]. Together, these results indicate that common marmosets are indeed susceptible to SARS-CoV-2, but their mild and inconsistent disease course, and apparent lack of a neutralizing antibody response, limits their usefulness as a good preclinical NHP model.

The susceptibility of NHPs to SARS-CoV-2 establishes them as useful pre-clinical animal models to study SARS-CoV-2, and they are currently being used extensively to gain insights into SARS-CoV-2 pathogenesis and for the development of countermeasures [207,208,209,210,211,212]. Moreover, the evolutionary relationship between humans and NHPs in terms of physiology, immunity, and pharmacodynamics provides an important benefit for using this model compared to other models such as hamsters, mice, or ferrets. However, the cost, inherent logistical difficulties, and ethical concerns of using NHPs as research subjects limits their usefulness to most researchers. It is important to note that each of the NHP species tested also exist as wild populations, i.e., these wild animals are also susceptible to natural SARS-CoV-2 infection. Moreover, other wild primates should be considered susceptible, which has been demonstrated by the infection of several gorillas held in a zoo that exhibited respiratory signs upon infection [137]. SARS-CoV-2 would likely transmit rapidly through closely interacting social groups of primates; therefore, caution should be taken at interfaces between humans and wild and captive non-human primates, particularly for critically endangered populations [213].

#### 2.2.11. Transgenic Laboratory Mice

Non-transgenic laboratory mice (Mus musculus) are not susceptible to SARS-CoV-2 infection with ancestral strains due to an incompatibility between SARS-CoV-2 and the mouse ACE2 receptor [1,142,214,215]. However, several transgenic mouse models have been utilized or developed that express the human ACE2 receptor (hACE2) and were found to be capable of becoming productively infected with SARS-CoV-2 [142,143,144,145,146,147,148,149,215,216].

Two different transgenic mouse lines were generated that expressed hACE2 under the mouse ACE2 promoter and were experimentally infected with SARS-CoV-2 [142,148]. Both transgenic mouse lines resulted in ~10% body weight loss after SARS-CoV-2 infection and developed pathological changes in the lungs, with interstitial pneumonia developing by 3 to 5 DPC. Infectious SARS-CoV-2 virus was isolated from the lungs of these mice from 1–5 DPC. Additionally, a mouse model previously developed for the expression of hACE2 under the expression of a ciliated lung specific HFH4/FOXJ1 promoter were also susceptible to SARS-CoV-2 infection [217]. Infection of these transgenic HFH4-hACE2 mice with SARS-CoV-2 [144] mostly results in a relatively mild, subclinical outcome, with SARS-CoV-2 RNA detected in lungs from 1–7 DPC, and an immune response that protects mice from reinfection. However, a significant proportion of the HFH4-hACE2 mice suffered fulminant disease upon SARS-CoV-2 infection, characterized by severe interstitial pneumonia, detection of infectious virus in the lung and, importantly, in brain tissue on 7 DPC, resulting in death around 8 DPC [144]. Similarly, transgenic mice had previously been developed for SARS-CoV research in which hACE2 expression was driven by the epithelial cell cytokeratin-18 (K18) [218]. Experimental infection of these K18-hACE2 transgenic mice with SARS-CoV-2 consistently resulted in a severe dose-dependent clinical disease, with marked weight loss, a range of clinical signs, including respiratory distress, ruffled fur, hunched posture, and lethargy, and mortality that occurred around 5 to 7 DPC [143,145,146,147,149,216]. Severe lung pathology is characteristic for the SARS-CoV-2 infected K18-hACE2 mice [143,145,146,147,149,216], along with virus detected in upper and lower respiratory tract samples [143,146,147,149,216]. Additionally, SARS-CoV-2 infection in the brain of K18-hACE2 mice is common, with increasing viral titers over the infection period, and is likely responsible for the lethality of SARS-CoV-2 infection in these mice [146,147,149,216]. Lastly, an adenovirus-based system was used to transduce different strains of laboratory mice, causing them to express hACE2 (AdV-hACE2) which allows SARS-CoV-2 susceptibility in a range of genetic backgrounds with differences in clinical disease progression based on the specific mouse strain used [147,215,219].

In addition, SARS-CoV-2 has been adapted, after several passages, to bind murine ACE2 and infect non-transgenic mice [138,139]. Moreover, it has been shown that non-transgenic laboratory mice are susceptible to SARS-CoV-2 variants containing the N501Y polymorphism in the S gene [140,141]. These mouse-permissive SARS-CoV-2 strains, therefore, provide additional tools to study SARS-CoV-2 infection in murine models.

Together, the combination of multiple transgenic hACE2-expressing mouse models under various promoters, adenovirus-based methods for transduction of multiple mouse strains with hACE2, mouse-adapted SARS-CoV-2 strains, and the susceptibility of mice to emerging VOC has resulted in laboratory mice becoming highly useful models for SARS-CoV-2 infection studies and efficacy testing of vaccines and therapeutics [220,221,222,223,224].

## 3. Summary and Conclusions

The ongoing COVID-19 pandemic is clearly the most consequential public health crisis of the 21st century. The scope of the challenge appears insurmountable and managing the pandemic or controlling and eradicating the disease will require unprecedented multidisciplinary cooperation. A clear understanding of the host range of SARS-CoV-2 is central to predicting the evolving disease ecology of the virus, and to anticipate complications that could alter the pandemic landscape. Evidence regarding SARS-CoV-2 susceptibility in domestic pets, farmed animals, wild animals, and laboratory model species is therefore necessary to coordinate research objectives and to inform public health policy. Research to date indicates that SARS-CoV-2 has an exceptionally broad host range and can infect many different mammalian species with a wide spectrum of disease manifestations.

A number of predictions were made regarding SARS-CoV-2 susceptibility in different animal species using in silico structure-guided analysis of the ACE2-RBD interface [185,225,226,227,228,229] and in vitro experiments in cultured cells derived from or expressing receptors from different animal species [230,231,232]. While informative, several inconsistencies between in silico/in vitro predictions and in vivo susceptibility have been observed, including predictions of susceptibility for pigs and cattle and resistance for ferrets, mink, and raccoon dogs. In vivo susceptibility, therefore, clearly involves a higher level of complexity, including ACE2 expression patterns, expression of cofactors, and immune responses of the host, that are unable to be recapitulated using in vitro or in silico models of infection. However, in silico and in vitro studies will continue to be important, particularly for assessing emerging SARS-CoV-2 variants.

Among domesticated species, cats, hamsters, ferrets, mink, raccoon dogs, and rabbits are susceptible to a productive SARS-CoV-2 infection and are all capable of transmitting the virus to naïve animals. Evidence suggests that dogs, cattle, and pigs are marginally susceptible to SARS-CoV-2 infection and are not suitable amplifying hosts. Chickens and several other poultry species are wholly resistant to SARS-CoV-2 infection. Among wild/peridomestic animals, deer mice, woodrats, skunks, otters, and white-tailed deer have been clearly shown to be susceptible to SARS-CoV-2 infection. Fruit bats are also susceptible but exhibit limited replication with inefficient transmission compared to other species. Tree shrews may also fit into this category, but the extent of their susceptibility will require additional investigation. Squirrels, prairie dogs, raccoons, and big brown bats appear to be resistant. House mice are clearly resistant to ancestral SARS-CoV-2 strains; however, their susceptibility to isolates carrying the N501Y polymorphism present in emerging SARS-CoV-2 VOCs enhances their status as potential carriers. Several species of NHPs have been shown to be susceptible to SARS-CoV-2, which has implications for wild primate populations.

Several species have now been identified as useful or potential pre-clinical models of SARS-CoV-2 infection. NHPs will continue to be important for pre-clinical studies, although costs and ethical concerns may limit their usefulness. Laboratory mice are also informative models, but the requirement for susceptible hACE2 transgenic mice or mouse-adapted/N501Y variant SARS-CoV-2 strains presents some limitations. Syrian golden hamsters have emerged as possibly the best model organism to study SARS-CoV-2 infection, as they consistently exhibit clear viral infection markers and clinical signs and are relatively easy to handle and house in biocontainment facilities. Dwarf hamster species may be used as an alternative as they present a similar disease progression compared to Syrian golden hamsters. Roborovski dwarf hamsters should be investigated in more detail based on their severe, fatal disease response to SARS-CoV-2 infection. Deer mice may also be a useful alternative to hamsters due to their genetic diversity compared to inbred rodents, although accessibility and scale up will likely be an issue. Ferrets are also useful models for SARS-CoV-2 infection, although the clinical signs observed have not always been consistent between studies. Cats may also be useful models, but the general absence of clinical signs, ethical concerns regarding their status as companion animals, and difficulties with handling and scale up presents limitations. Rabbits could also be used as SARS-CoV-2 infection models, however the absence of clinical signs and the requirement of high virus doses for productive infection are potential drawbacks. Highly susceptible white-tailed deer have also proven to be useful models; however, scale up, housing, and handling again provide complications to their routine use. Although tree shrews do not appear to be reliably susceptible to SARS-CoV-2, the unique manifestation of viral replication in non-respiratory organs, e.g., pancreas, may be worth further investigation. Overall, the broad host range of SARS-CoV-2 provides several options for pre-clinical animal models, with different benefits and limitations, which can be reliably used for basic research and the development of effective countermeasures.

There has been significant concern throughout the pandemic regarding the potential for humans to infect companion animals, potentially causing illness in pets and establishing new reservoirs. Instances of natural human-to-animal infection (reverse zoonosis) have been demonstrated for cats, ferrets, and dogs, although no evidence to date has been shown for hamsters. At the present time, the potential for infected pets to become amplifying hosts or a SARS-CoV-2 reservoir species appears to be low, and concerns regarding pet health in terms of SARS-CoV-2 is rather small. However, precautions should still be taken to limit exposure of naïve individuals to cats, dogs and ferrets living in COVID-19-affected households based on their ability to efficiently transmit the virus. Abandoning potentially infected pets should be highly discouraged, especially since it could facilitate further spread to outdoor domestic and wild animals or group-housed shelter populations. Pet hamsters should be monitored closely for clinical signs of infection in COVID-19-affected households, and infected humans should avoid handling hamsters and practice common sense to avoid human-to-hamster infection or vice versa. Continued surveillance of pets in areas with high SARS-CoV-2 transmission rates, and sequencing of any collected viral strains, would provide a more complete assessment of the involvement of pets in the pandemic.

Fortuitously, intensively farmed cattle, pigs, and chickens are rather resistant to SARS-CoV-2 infection. Susceptibility in any of these species would severely alter the pandemic landscape, considering the large number of these animals that are raised near each other and near humans; it would also have severe implications on global food security. Poultry appear to be completely resistant to SARS-CoV-2 infection, but cattle and pigs show minimal susceptibility with some evidence of viral replication and immune response. Cohorts used in experimental cattle and pig studies were small, and susceptibility could increase in different breeds and ages. Moreover, novel SARS-CoV-2 variants or recombinant strains with widely circulating swine and bovine coronaviruses could potentially change this situation. An in vitro study indicated that SARS-CoV-2 can replicate in ex vivo respiratory tissue from sheep [233], although a recent study suggests resistance in sheep to natural SARS-CoV-2 infection [234]. Experimental studies of SARS-CoV-2 infection in sheep—and other farmed species, namely goats, equids, and camelids—should be pursued.

The clear susceptibility of mink has resulted in arguably the most significant event involving the human–animal interface during the SARS-CoV-2 pandemic. Outbreaks in mink farms worldwide are of significant concern for several reasons, including; (i) outbreaks occurred via human-to-mink transmission in spite of guidelines mandating use of personal protective equipment, (ii) outbreaks spread rapidly through the farms, propelled by the proximity of the animals and efficient aerosol transmission, (iii) SARS-CoV-2 adapted to mink, resulting in spike RBD mutations that altered the neutralizing capability of convalescent human sera, (iv) mink successfully infected humans working on farms with mink-derived SARS-CoV-2 variants, and (v) insecure boundaries surrounding mink farms resulted in SARS-CoV-2 infection of cats and wild mink. These events are concerning and indicate that a normally solitary wild species is capable of sustaining SARS-CoV-2 outbreaks when housed in large numbers for commercial purposes. Detailed epidemiological investigations of mink outbreaks should be continued and improved biosecurity and monitoring protocols should be implemented for mink farms throughout the pandemic. Overall, the mink farm outbreaks raise awareness to the importance of zoonotic/reverse zoonotic transmissions of SARS-CoV-2 and highlights the importance of a One Health approach to the pandemic.

Raccoon dogs, ferrets, and rabbits represent similar opportunities for reverse zoonosis events but are currently of lesser concern. Raccoon dogs are farmed for their fur in conditions comparable to mink but appear to be less susceptible to infection, and thus far no outbreaks have been reported. Ferrets are not generally housed in large numbers; therefore widespread transmission is unlikely. Conversely, rabbits may be housed in large numbers in farms for meat production, but their susceptibility appears to be lower than that of mink and their ability to transmit the virus is unknown. Regardless, raccoon dogs, rabbits, and ferrets should be subjected to active monitoring during the pandemic, and humans in close contact should adhere to reasonable biosecurity measures.

The established susceptibility of several wild species to SARS-CoV-2 is of significant concern and these species could become secondary reservoirs of the virus. Large numbers of susceptible deer mice, woodrats, and white-tailed deer are present over large areas of North America. Each of these species have the potential to become SARS-CoV-2 reservoirs as evidenced by their role as carriers of other bacterial and viral diseases that infect humans. Recent evidence suggests that white-tailed deer may have already become a reservoir species for SARS-CoV-2, although additional information is needed to understand the full scope and implications of the situation. In addition, skunks, wild mustelids, and fruit bats have all been shown to be susceptible to SARS-CoV-2 infection. Wild populations of primates, *Cricetidae* rodents, felids, mustelids, and cervids, many of which are vulnerable or endangered, should be considered susceptible and efforts to avoid or practice appropriate biosecurity is warranted. Moreover, active surveillance of wild species that are potentially susceptible should be performed to understand the full extent of SARS-CoV-2 spread among wild animal species.

The emergence of zoonotic diseases like SARS-CoV-2, is a complex process whereby various pressures allow a virus to mutate and adapt to new hosts and environments. In recent times, an estimated 75% of novel emerging diseases in humans have been zoonotic in origin [235]. The combination of large-scale intensive farming, wet markets and agricultural fairs housing large numbers of different animal species, widespread trade of exotic animals, human encroachment on wild habitat due to agricultural and industrial needs or urbanization, increased global travel, and unknown consequences of global climate change all have significant implications that affect the human–animal interface, and make the emergence of novel zoonotic diseases inevitable [236,237,238]. Informed policy on these matters should always consider the potential for the emergence of zoonotic diseases. Such policies will require a One Health approach combined with extensive outreach into human populations that live and work at the human–animal interface. Moreover, human-to-human contact remains the ultimate driver of the current pandemic, and continued vigilance to manage or eradicate SARS-CoV-2 in the human population will ultimately lessen the risks associated with a reverse zoonotic spillover to susceptible animals.
